# Hot Deformation Behavior and Microstructure Evolution of a Novel Near-α Titanium Alloy with Initial Lamellar Microstructure During Hot Compression

**DOI:** 10.3390/ma19173749

**Published:** 2026-09-03

**Authors:** Xiaojuan Jiang, Lili Wu, Tao Sun, Jie Zhou

**Affiliations:** 1 School of Mechanical Engineering, Chongqing Industry Polytechnic University, Chongqing 401120, China; jiangxj@cqipu.edu.cn (X.J.); wulilishini@126.com (L.W.); 2 College of Mechanical and Vehicle Engineering, Chongqing University, Chongqing 400030, China; 3 College of Materials Science and Engineering, Chongqing University, Chongqing 400045, China; zhouj_cqu@163.com

**Keywords:** Ti65 titanium alloy, hot deformation, constitutive model, hot processing map, microstructure evolution

## Abstract

The hot deformation behavior and microstructure evolution of a novel near-α Ti65 titanium alloy with an initial lamellar microstructure were investigated by isothermal compression. Compression tests were conducted at 950–1010 °C in the α + β phase region and 1050–1110 °C in the β phase region, with strain rates of 0.01–10 s^−1^ and deformation amounts of 30–75%. The flow behavior, strain-compensated Arrhenius constitutive model, processing map and microstructural evolution were systematically analyzed. The results indicate that the flow stress decreases with increasing temperature and decreasing strain rate, while flow softening is more pronounced in the α + β region than in the β region. The apparent activation energies are 1050.27 kJ/mol for the α + β region and 203.51 kJ/mol for the β region, indicating distinct deformation mechanisms. The established constitutive models exhibit high prediction accuracy, with R and AARE values of 0.98 and 5.97% in the α + β region and 0.99 and 4.29% in the β region, respectively. Processing-map analysis identifies two instability domains at high strain rates: 990–1020 °C/3.5–10 s^−1^ in the upper α + β region and 1060–1110 °C/1.65–10 s^−1^ in the β region. Microstructural observations reveal that dynamic spheroidization of lamellar α dominates deformation in the α + β region, whereas dynamic recovery accompanied by limited β dynamic recrystallization occurs in the β region. Increasing deformation amount at 980 °C and 0.01 s^−1^ promotes α-lamella fragmentation, spheroidization, grain refinement and texture weakening. The maximum pole density decreases to 6.23 mrd at a high deformation amount. By directly correlating strain-dependent processing-map characteristics with quantitative microstructural and crystallographic evolution, this work provides a microstructure-based basis for optimizing the hot-working window of Ti65 alloy with an initial lamellar microstructure.

## 1. Introduction

With the continuous pursuit of higher thrust-to-weight ratios and improved fuel efficiency in advanced aero-engines, structural materials used in compressor discs and blades are required to retain high specific strength, creep resistance and microstructural stability under increasingly severe thermal–mechanical conditions [1,2]. Near-α titanium alloys, which contain a high fraction of α-stabilizing elements and limited β stabilizers, have therefore attracted sustained attention because they offer a desirable combination of low density, excellent high-temperature strength, good fatigue resistance and oxidation/creep resistance compared with many conventional titanium alloys [3,4,5]. Representative alloys such as IMI834, Ti-6242S and Ti60 have been successfully developed for elevated-temperature aero-engine applications; however, the long-term service capability of most traditional high-temperature titanium alloys is generally limited to approximately 600 °C, beyond which thermal exposure may accelerate α_2_-Ti_3_Al ordering silicide precipitation, microstructural coarsening and consequent degradation of ductility and damage tolerance [1,6]. From an alloy-design perspective, these near-α alloys represent a progressive increase in compositional complexity for elevated-temperature applications. Ti-6242S is based primarily on the Ti-Al-Sn-Zr-Mo-Si system and is typically used at temperatures of approximately 500–550 °C, whereas IMI834 additionally contains Nb, Si and a small amount of C and has a service capability approaching 600 °C. Ti60 was subsequently developed with a composition similar to IMI834 but with the introduction of Ta and an increased Si content to further improve high-temperature strength and creep resistance. Ti65 extends this alloying strategy through a multi-component Ti-Al-Sn-Zr-Mo-Nb-Ta-Si-W-C system, in which W is additionally introduced together with Ta and the balance among the β-stabilizing refractory elements is further adjusted. Ta and W preferentially partition to the β phase in Ti65, with W exhibiting particularly strong β-partitioning behavior. The combined addition of these refractory elements has been reported to contribute to solid-solution strengthening, thermal stability, and creep/durability at elevated temperatures, while W can also contribute to grain refinement and high-temperature strength. This more highly alloyed design enables Ti65 to target a higher service-temperature range of approximately 600–650 °C but also makes its phase constitution and hot deformation response highly sensitive to thermomechanical-processing parameters. Nevertheless, the superior service performance of such multi-component alloys can only be realized through precise thermomechanical processing. Understanding the hot deformation response of Ti65 is thus essential for controlling microstructure evolution and establishing robust forming routes for high-performance aero-engine components.

For titanium alloys with an initial lamellar microstructure, hot deformation is not only a macroscopic plastic-flow process but also a complex microstructural reconstruction process involving the coordinated evolution of α lamellae, retained β layers, colony boundaries and prior β grains [7,8,9]. Compared with equiaxed or bimodal microstructures, lamellar colonies usually exhibit pronounced deformation heterogeneity because the activation of basal, prismatic and pyramidal slip systems in the α phase is strongly dependent on the orientation of α lamellae relative to the loading direction, whereas the thin β films between α plates can accommodate strain through dynamic recovery and local recrystallization [6,10]. Therefore, the flow behavior during hot compression is highly sensitive to deformation temperature, strain rate and strain. Increasing temperature or decreasing strain rate generally reduces flow stress by promoting dislocation climbing, interface migration, and diffusion-controlled softening, while excessive strain rates tend to intensify work hardening and localized deformation due to insufficient dynamic restoration [8,11]. Stacking-fault energetics also play an important role in determining the competition between dislocation recovery and recrystallization during hot deformation. For FCC metals, a relatively high stacking-fault energy (SFE) reduces the separation of Shockley partial dislocations and facilitates dislocation constriction and cross-slip, thereby promoting dynamic recovery. For example, during hot working, aluminum with a relatively high SFE generally exhibits recovery-dominated restoration, whereas copper with a lower SFE shows less effective recovery and a stronger tendency toward recrystallization under comparable deformation conditions [12]. For HCP α-Ti, however, this effect is more appropriately described by the generalized stacking-fault energy (GSFE), which depends strongly on the active basal, prismatic and pyramidal slip systems rather than being represented by a single SFE value. Previous first-principles studies have demonstrated that alloying elements can modify the GSFE and dislocation-slip barriers of α-Ti, thereby affecting dislocation mobility and the balance between dislocation storage and restoration [13,14]. Therefore, stacking-fault energetics constitute an additional atomic-scale factor influencing the recovery behavior of the multi-component Ti65 alloy, together with deformation temperature, strain rate, phase constitution and α/β interfacial processes.

In the α + β phase region, lamellar titanium alloys often show obvious flow softening after the peak stress, which is mainly associated with lamellar kinking, colony rotation, and the dynamic globularization of α lamellae [15,16,17]. During dynamic globularization, dislocations, subboundaries, kink bands or shear bands are first introduced within α lamellae, producing local misorientation gradients and high-energy defects. These defects promote α/β interfacial instability, boundary splitting, and β phase penetration, eventually leading to fragmentation, spheroidization, and the coarsening of α lamellae [6,17,18]. EBSD studies on titanium alloys have shown that deformation-induced low-angle boundaries may progressively evolve into high-angle boundaries through subgrain rotation, a process that can be associated with continuous dynamic recrystallization (CDRX). However, the LAB-to-HAB transition is not by itself a unique criterion for identifying CDRX, because similar boundary evolution may also accompany dynamic recovery, lamellar subdivision, and dynamic spheroidization. For Ti65 specifically, recent studies have demonstrated that the dominant restoration mechanism strongly depends on the phase constitution, initial microstructure, and deformation conditions. CDRX has been reported to dominate in the α + β phase region under certain hot deformation conditions, whereas dynamic recovery and discontinuous dynamic recrystallization (DDRX) become more important in the β single-phase region [19]. Other studies on Ti65 with different initial microstructures have identified dynamic spheroidization and boundary splitting of lamellar α as the principal softening mechanisms in the α + β region [5]. Therefore, the evolution of low- and high-angle boundaries should be interpreted together with grain morphology, intragranular orientation gradients, and other microstructural evidence when distinguishing among recovery, recrystallization, and spheroidization mechanisms. In addition to microstructural characterization, processing-map analysis has been widely used to identify suitable hot-working domains for titanium alloys. Based on the dynamic materials model, a processing map combines the power dissipation efficiency map with the flow-instability map, thereby correlating deformation parameters with possible microstructural mechanisms and unsafe deformation regions [20,21]. Domains with high power dissipation efficiency are generally associated with dynamic recovery, dynamic recrystallization, phase transformation or globularization, whereas instability domains may correspond to adiabatic shear bands, flow localization, cracking or severe microstructural heterogeneity [22,23,24]. Therefore, processing maps provide an effective approach for optimizing hot deformation parameters, but their interpretation should be verified by quantitative microstructural evidence rather than relying only on flow-stress data [25]. Previous studies have provided important insights into the hot deformation behavior of Ti65 alloy from different perspectives. Zhang et al. investigated the microstructural and crystallographic evolution of Ti65 with a lamellar microstructure and revealed strain-dependent α-lamella fragmentation, spheroidization, texture evolution, and β phase recrystallization during hot compression [26]. More recently, Sun et al. established constitutive models and analyzed the deformation mechanisms of Ti65 with an initial bimodal microstructure, demonstrating pronounced differences in flow softening and restoration mechanisms between the α + β and β phase regions [5]. In addition, Zhu et al. constructed processing maps for Ti65 and combined them with finite-element simulations to optimize multi-directional forging parameters [20]. However, these studies mainly addressed microstructural mechanisms, constitutive prediction, or process optimization separately, and a systematic correlation among processing-map characteristics, flow instability, and quantitative microstructural evolution for Ti65 with an initial lamellar microstructure remains insufficiently established.

Although Ti65 has been recognized as a promising high-temperature near-α titanium alloy, systematic understanding of its hot deformation response from an initial lamellar microstructure is still limited. Therefore, the present work focuses on the coupled relationship among flow behavior, constitutive response, processing-map evolution, and microstructural mechanisms of lamellar Ti65 alloy. In particular, strain-dependent processing maps are correlated with representative microstructures from stable and unstable deformation domains, while EBSD analyses are used to quantify boundary evolution, crystallographic misorientation, and texture weakening with increasing deformation. This integrated approach enables the processing-map domains to be interpreted on the basis of experimentally observed α-lamella fragmentation and dynamic spheroidization in the α + β region and dynamic recovery accompanied by limited β dynamic recrystallization in the β region. The results provide a microstructure-based basis for identifying robust hot-working windows for Ti65 alloy with an initially lamellar microstructure. Cylindrical specimens with dimensions of Φ8 × 12 mm were subjected to isothermal hot compression. The tests were conducted at 950, 980 and 1010 °C in the α + β region and at 1050, 1080 and 1110 °C in the β region, with strain rates of 0.01, 0.1, 1 and 10 s^−1^. Deformation amounts of 30%, 45%, 60% and 75% were selected to reveal strain-dependent microstructure evolution. These results provide a microstructure-based basis for selecting and optimizing hot-working parameters for Ti65 alloy with an initial lamellar microstructure.

## 2. Materials and Experimental Methods

### 2.1. Materials and Hot Compression Experiments

**Figure 1:**
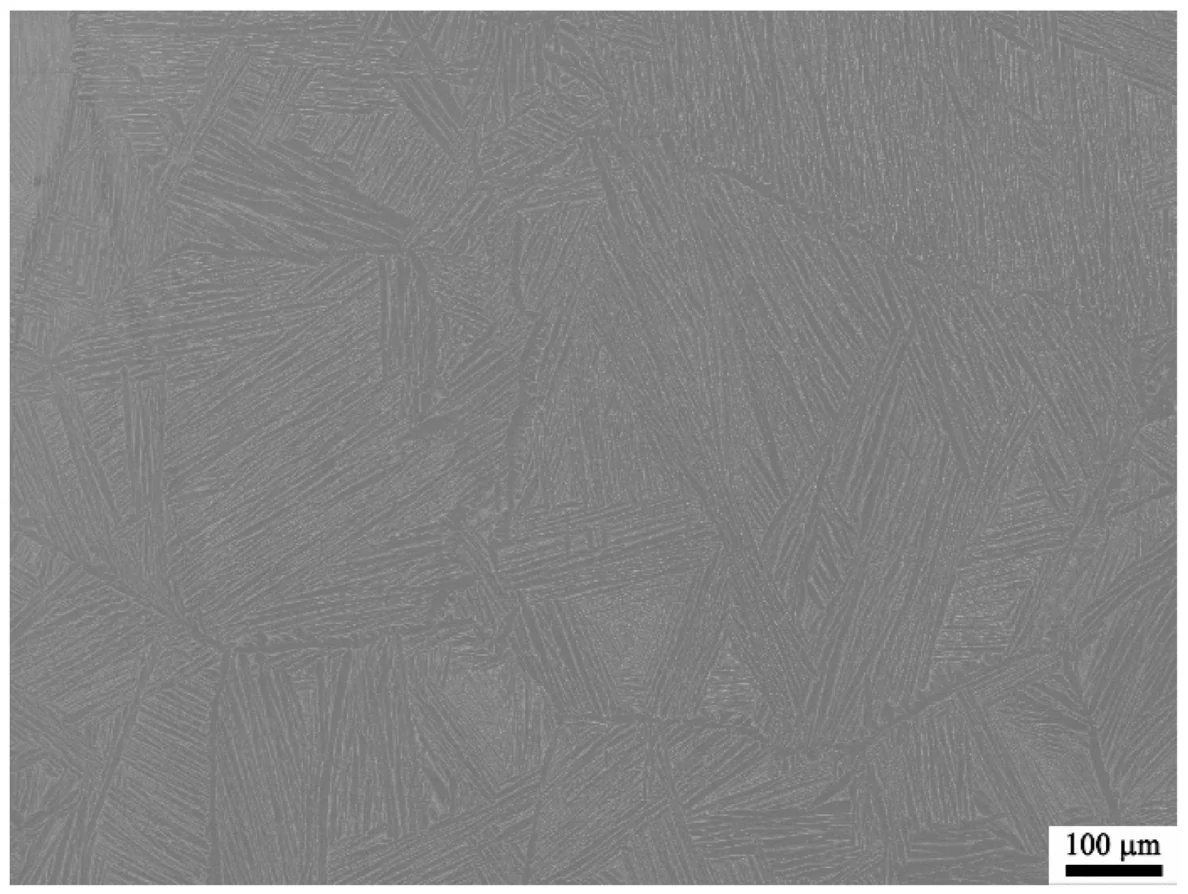
Initial lamellar microstructure of Ti65 alloy.

The material investigated in this work was a newly developed near-α high-temperature titanium alloy, designated as Ti65 in China. The alloy was supplied in the as-forged condition after thermomechanical processing in the β single-phase region and subsequent cooling to room temperature, and it exhibited an initial lamellar microstructure, as shown in Figure 1. To reveal the initial microstructure, the metallographically polished specimen was chemically etched for approximately 10 s using a solution of HF, HNO_3_ and H_2_O with a volume ratio of 1:3:96. The chemical composition of the as-received Ti65 alloy was determined using an X-ray fluorescence spectrometer (XRF-1800, Shimadzu Corporation, Kyoto, Japan), and the measured composition is listed in Table 1. The α phase of Ti65 possesses a hexagonal close-packed (HCP) crystal structure, whereas the β phase has a body-centered cubic (BCC) structure. Among the alloying elements in Ti65, Al and the interstitial element C act primarily as α phase stabilizers, while Mo, Nb, Ta and W are β phase stabilizers. Sn and Zr are generally regarded as near-neutral alloying elements in conventional titanium alloys; however, Zr has been reported to exhibit a weak β-stabilizing tendency in Ti65 owing to its preferential partitioning into the retained β phase [27]. Si is mainly introduced to improve the high-temperature properties and participates in silicide formation rather than acting as a primary phase-stabilizing element. The β-transus temperature was determined using a metallographic heat-treatment method. Six heat-treatment temperatures of 1025, 1030, 1035, 1040, 1045, and 1050 °C were selected at intervals of 5 °C. The specimens were held at each temperature for 20 min and then quenched to room temperature. After metallographic preparation, the presence and amount of residual primary α phase were examined, as shown in Figure S1. The β-transus was evaluated from the progressive decrease and eventual disappearance of the residual primary α phase with increasing temperature. Specifically, the transformation-temperature interval was identified between the highest temperature at which residual primary α was still detectable and the lowest temperature at which the primary α phase had completely disappeared. Based on this criterion, the β-transus temperature of the investigated Ti65 alloy was determined to be approximately 1045 °C. According to this temperature, the selected deformation temperatures were divided into the α + β phase region and the single β phase region.

Cylindrical compression specimens with a diameter of 8 mm and a height of 12 mm (height-to-diameter ratio of 1.5) were machined from the as-forged billet by electrical discharge machining. The specimen dimensions were selected based on commonly adopted geometries for isothermal hot compression testing and the dimensional requirements of the Thermecmastor-Z thermal simulator, rather than a specific standardized specimen geometry [25,28]. This geometry provides sufficient specimen stability during compression and an adequate central region for subsequent microstructural characterization. The axial direction of each specimen was parallel to the forging direction of the billet. To reduce friction during compression and improve the reliability of the uniaxial deformation condition, tantalum foils and graphite lubricant were placed between the specimen and the anvils. A thermocouple was welded at the mid-height of each specimen to monitor and control the actual deformation temperature. Before heating, argon gas was introduced into the chamber to protect the specimen from severe oxidation. Isothermal hot compression tests were carried out using a Thermecmastor-Z thermal simulator. The specimens were heated to the target deformation temperature at a heating rate of 10 °C/s and then held for 300 s to ensure temperature homogenization before deformation. The 300 s holding period was employed to establish a uniform and stable temperature distribution before compression. It should be noted that the “initial lamellar microstructure” in this work refers to the as-received microstructure prior to heating. During holding in the α + β phase region, thermal exposure may cause redistribution of the α/β phase fractions, partial dissolution of lamellar α, and limited thermally activated interfacial evolution. At temperatures above the measured β-transus (~1045 °C), the lamellar α phase progressively dissolves into the β matrix during heating and holding, and deformation therefore occurs in the β single-phase state. Thus, the microstructures observed after compression reflect the combined effects of the prescribed thermal exposure and subsequent deformation.

**Figure 2:**
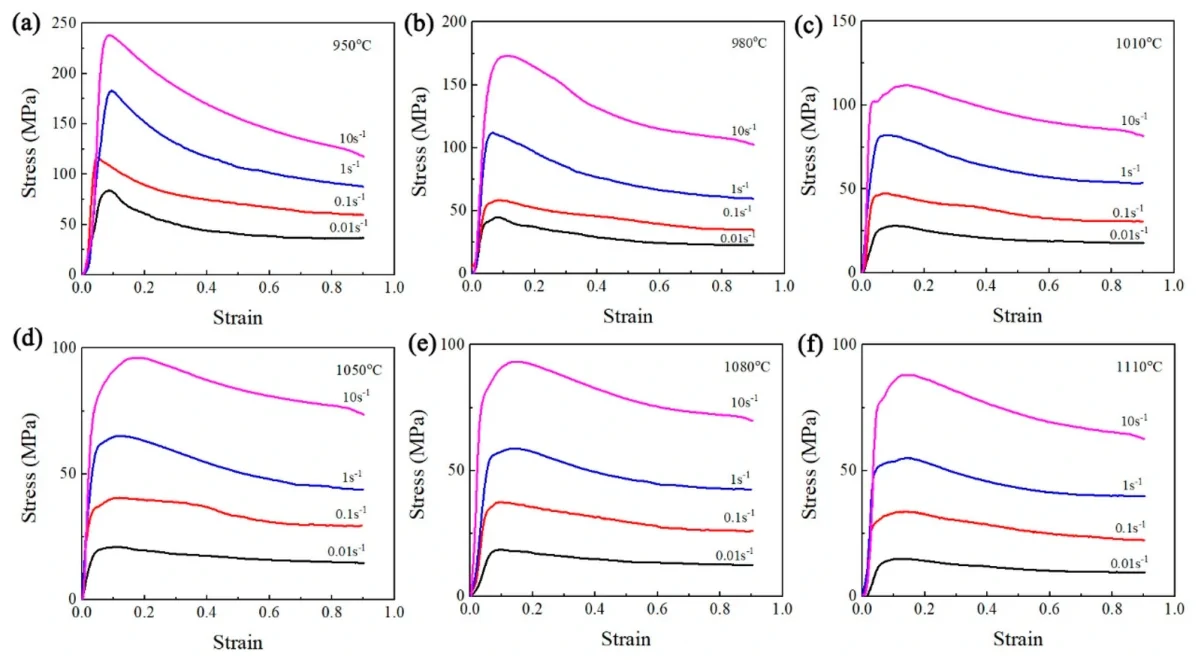
True stress–strain curves of lamellar microstructure under different hot compression conditions of Ti65 alloy: (**a**) 950 °C, (**b**) 980 °C, (**c**) 1010 °C, (**d**) 1050 °C, (**e**) 1080 °C, (**f**) 1110 °C.

Compression experiments were performed at 950, 980 and 1010 °C in the α + β phase region and at 1050, 1080 and 1110 °C in the β phase region. For each temperature, four strain rates of 0.01, 0.1, 1 and 10 s^−1^ were employed. To reveal the strain-dependent microstructure evolution, interrupted compression tests were conducted at different deformation amounts of 30%, 45%, 60% and 75%. For each temperature–strain-rate condition, one valid compression test was used to construct the flow-stress curve. Therefore, the curves shown in Figure 2 represent individual tests rather than averages of repeated measurements, and statistical standard deviations of flow stress are not available for the complete deformation matrix. In this work, the deformation amount refers to the reduction in specimen height and is defined as ($D\;=\;(h_{0}\;-\;h)/h_{0}$), where *h*_0_ and *h* are the initial and instantaneous specimen heights, respectively. The corresponding compressive true strain is expressed as $\varepsilon \;=\;\ln\left(h_{0}/h\right)\;=\;-ln(1\;-\;D)$, with *D* expressed as a decimal fraction. The deformation amounts were used to define the interrupted compression conditions for microstructural characterization, whereas the true strains employed in the constitutive-model and processing-map analyses were selected directly from the continuous true stress–true strain curves. After compression, the deformed specimens were immediately quenched to retain the high-temperature deformation microstructures for subsequent characterization.

### 2.2. Microstructure Characterization

After hot compression, the deformed specimens were sectioned along the compression direction, and the central region with the highest deformation degree was selected for microstructural characterization. For metallographic and SEM observations, the samples were sequentially ground using SiC abrasive papers from 400 to 3000 grit, followed by mechanical polishing with diamond suspension until a mirror-like surface was obtained. The polished surfaces were then etched for approximately 10 s using a mixed solution of HF, HNO_3_ and H_2_O with a volume ratio of 1:3:96 to reveal the grain boundaries, α/β phase interfaces and lamellar microstructural features. After etching, the samples were immediately rinsed with ethanol and dried using cold air. Metallographic observation was carried out to examine the overall microstructure, including α colonies and lamellar α morphology. SEM characterization was further performed using a JEOL-7800F field-emission scanning electron microscope (JEOL Ltd., Tokyo, Japan) to observe the detailed evolution of α lamellae and local spheroidization features under different deformation conditions.

For EBSD analysis, the mechanically polished specimens were further electropolished to remove the deformation layer introduced during mechanical preparation and to obtain a high-quality surface suitable for diffraction pattern acquisition. Electropolishing was conducted using an electrolyte composed of 5 vol.% perchloric acid, 35 vol.% n-butanol and 60 vol.% methanol at −35 °C and 30 V. The EBSD measurements were performed on the same JEOL-7800F scanning electron microscope equipped with an Oxford Instruments EBSD detector. An accelerating voltage of 20 kV was used, and the scanning step size was set to 0.2 μm according to the microstructural scale of the alloy. The analyzed area was approximately 200 × 150 μm^2^ for each EBSD map. The acquired EBSD data were processed using AZtecCrystal (Version 2.12) software. During post-processing, isolated zero-solution points were removed by nearest-neighbor interpolation, and noise reduction was conducted to improve the reliability of orientation analysis. For the grain-boundary analysis, boundaries with misorientation angles of 2–15° were classified as low-angle boundaries (LABs), whereas boundaries with misorientation angles ≥ 15° were classified as high-angle boundaries (HABs). Misorientations below 2° were excluded from the grain-boundary statistics to minimize the influence of orientation noise. Inverse pole figure maps, grain-boundary maps, and pole figures were used to characterize crystallographic orientation, boundary misorientation, and texture evolution of the deformed Ti65 alloy.

## 3. Results and Discussion

### 3.1. Flow Behavior Analysis

The true stress–strain curves obtained during high-temperature deformation provide an integrated response of the microstructural state and deformation characteristics of titanium alloys. In general, the flow behavior is strongly dependent on both the imposed deformation parameters and the initial microstructure of the material. Figure 2 presents the true strain–stress curves of the Ti65 alloy with an initial lamellar microstructure under different hot compression conditions. At the initial stage of deformation, the increase in flow stress can be attributed to strain hardening associated with the accumulation and interaction of dislocations [29]. Because direct dislocation characterization was not performed, this interpretation should be regarded as a mechanistic inference based on the observed flow response rather than as direct experimental evidence. Meanwhile, dynamic restoration mechanisms, such as dynamic recovery, dynamic recrystallization and globularization of the α phase, are still insufficiently activated at this stage [10,30]. With increasing strain, dynamic softening mechanisms, including dynamic recovery and the progressive fragmentation and spheroidization of lamellar α, become increasingly active and counteract the strain-hardening effect, eventually leading to a peak or a steady/softening flow response depending on the deformation condition.

At high strain rates, part of the plastic work can be converted into heat faster than it can be dissipated to the surroundings, resulting in a transient adiabatic temperature rise. Although the specimen temperature was monitored by a thermocouple welded at the mid-height surface, the deformation time at 10 s^−1^ is very short (approximately 0.09 s to a true strain of 0.9), and a local temperature rise in the specimen interior cannot be completely excluded. A first-order adiabatic estimate based on the measured stress–strain curves indicates that the theoretical upper-bound temperature rise at 10 s^−1^ is on the order of approximately 20–47 °C over the investigated temperature range, with the largest value occurring at the lowest deformation temperature. Because heat transfer to the anvils and surroundings was neglected in this estimate, the actual temperature rise is expected to be lower. Nevertheless, adiabatic heating may contribute to the apparent flow softening and flow instability observed under high-strain-rate conditions, particularly at relatively low temperatures.

**Figure 3:**
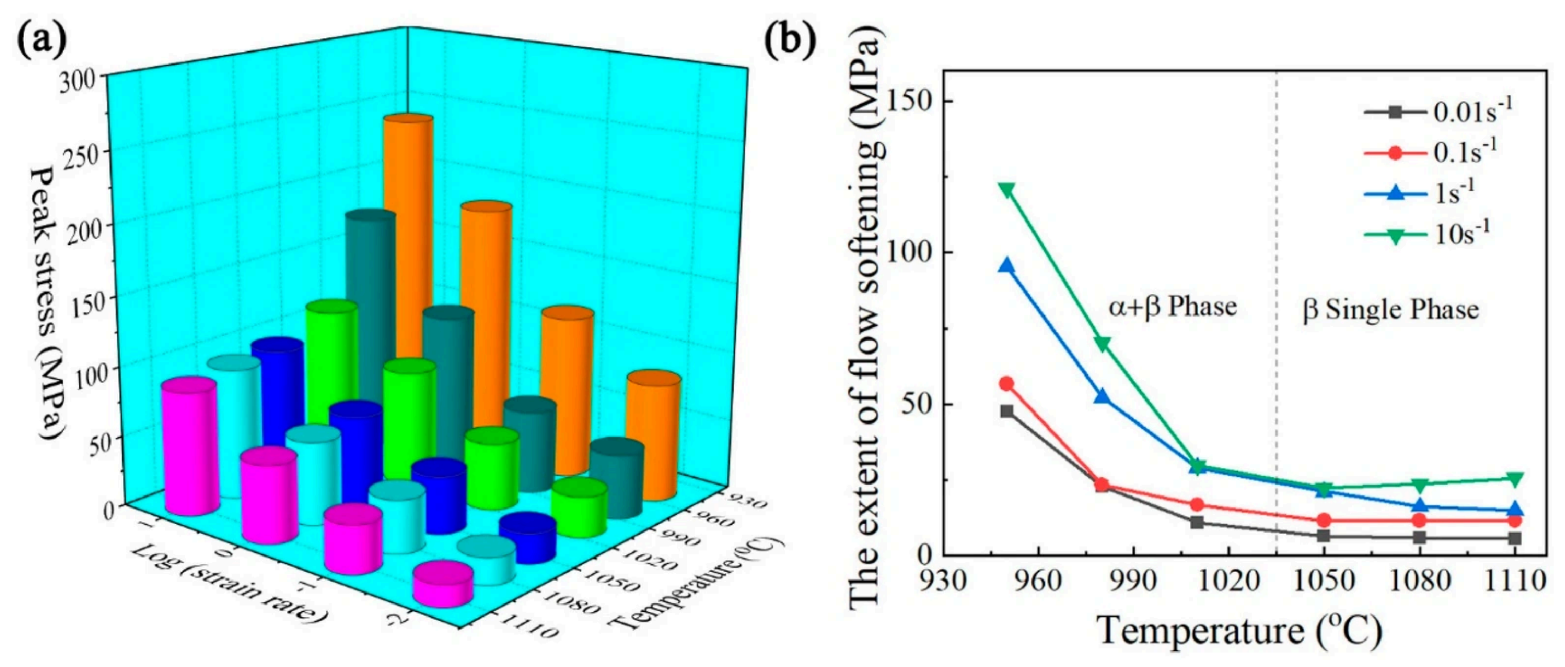
(**a**) Relationship between the peak stress and hot deformation parameters, (**b**) relationship between the extent of flow softening and deformation parameters.

The peak stress of the lamellar Ti65 alloy varies markedly with deformation temperature and strain rate, as summarized in Figure 3a. It can be seen that the peak stress decreases with increasing deformation temperature and decreasing strain rate. The decrease in peak stress with increasing deformation temperature and decreasing strain rate can be attributed to the competition between work hardening and thermally activated restoration processes. At elevated temperatures, enhanced atomic diffusion increases dislocation mobility and facilitates dislocation climb, rearrangement and annihilation, thereby promoting dynamic recovery and reducing the accumulated dislocation density. Meanwhile, enhanced interfacial mobility favors boundary migration and microstructural reconstruction during hot deformation. A lower strain rate provides sufficient time for these thermally activated processes to proceed at a given strain, whereas at higher strain rates, the rapid multiplication and accumulation of dislocations cannot be effectively compensated by dynamic restoration, resulting in stronger work hardening and higher flow stress [5]. Similar behavior has recently been reported for near-α titanium alloys, in which increasing temperature and decreasing strain rate promoted microstructural evolution, α-lamella spheroidization and dynamic restoration [11,31]. In addition, with increasing temperature in the α + β phase region of Ti65 alloy, the increasing fraction of the β phase, which possesses a BCC structure and more readily activated slip systems, improves deformation compatibility and further decreases the deformation resistance [4]. Therefore, the combined effects of enhanced dislocation restoration, interfacial migration, α-lamella spheroidization and temperature-dependent phase constitution account for the observed reduction in peak stress. Figure 3b further compares the degree of flow softening under different deformation conditions. The results show that the softening degree of the lamellar Ti65 alloy decreases as the temperature increases and the strain rate decreases. In addition, the flow-softening behavior in the α + β phase region is more pronounced than that in the single β phase region, suggesting that the deformation-induced evolution of lamellar α, such as kinking, fragmentation and spheroidization, plays an important role in the softening response during subtransus deformation.

### 3.2. Constitutive Model Construction

During hot deformation, the flow stress of metallic materials is governed by the combined effects of deformation temperature, strain rate and strain. Therefore, considerable efforts have been devoted to developing constitutive models that can quantitatively describe the relationship between flow stress and thermomechanical parameters [28]. For hot forming processes, an accurate constitutive equation is of great importance because it not only characterizes the deformation response of the material but also provides essential input data for predicting forming loads and conducting numerical simulations. Accordingly, the establishment of a reliable constitutive model is necessary for understanding and optimizing the hot-working behavior of the Ti65 alloy.

According to their theoretical basis and modeling strategy, constitutive models for hot deformation can generally be classified into three categories: phenomenological models, physically based models and data-driven models, such as artificial neural networks. Among them, phenomenological constitutive models have been widely used because of their relatively simple mathematical form and acceptable prediction accuracy [32]. For titanium alloys, the Arrhenius-type model expressed by a hyperbolic sine function is commonly employed to describe flow stress over a wide range of stress levels during hot deformation [33]. The relationship among strain rate, flow stress and deformation temperature can be expressed as follows:

(1) $$\dot{\varepsilon }\;=\;\left\{\begin{array}{c} A_{1}\sigma^{n_{1}}exp\left(-\frac{Q}{RT}\right)(\alpha \sigma \;<\;0.8)\\ A_{2}exp(\beta \sigma )exp\left(-\frac{Q}{RT}\right)(\alpha \sigma \;>\;1.2)\\ A[sinh(\alpha \sigma ){]}^{n}exp(\;-\;Q/RT) \end{array}\right.$$

**Figure 4:**
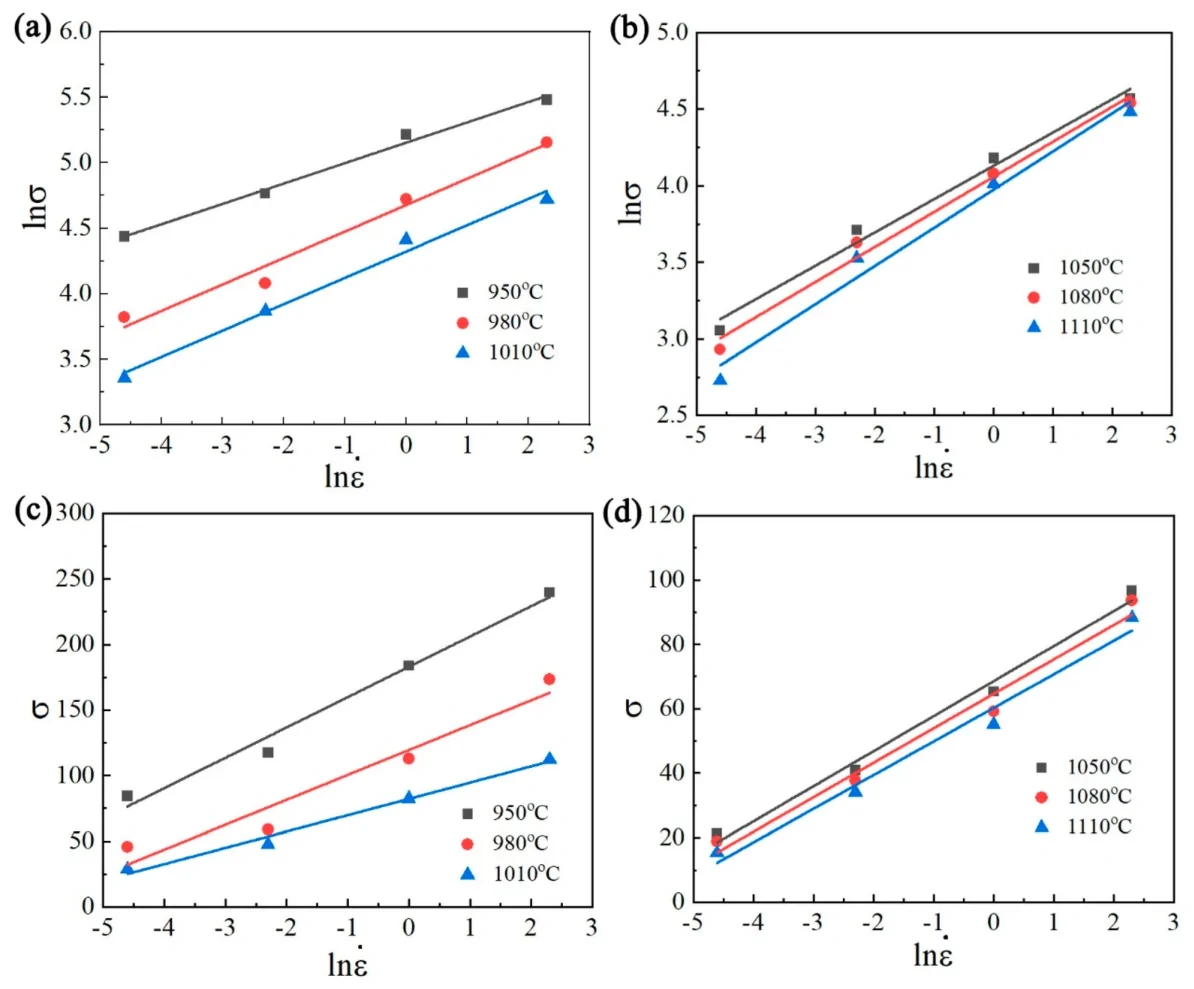
The correlation curves of lamellar microstructure: (**a**) ln*σ*-$\ln\dot{\varepsilon }$ in the α + β phase field, (**b**) ln*σ*-$\ln\dot{\varepsilon }$ in the β phase field, (**c**) *σ*-$\ln\dot{\varepsilon }$ in the α + β phase field, (**d**) *σ*-$\ln\dot{\varepsilon }$ in the β phase field.

In these equations, $\dot{\varepsilon }$, σ, *T*, *Q*, and R denote the strain rate, flow stress, absolute temperature, apparent activation energy for hot deformation, and universal gas constant, respectively. *A*_1_, *A*_2_, *A*, *n*_1_, *n*, α, and β are material-dependent constants, among which α is generally calculated as $\beta /n_{1}$. To facilitate the determination of the material constants involved in the above constitutive equations, the natural logarithm was taken on both sides of Equation (1), and the following linearized expressions were obtained:

(2) $$ln\dot{\varepsilon }\;=\;\left\{\begin{array}{l} lnA_{1}\;+\;n_{1}ln\sigma \;+\;(-Q/RT)\\ lnA_{2}\;+\;\beta \sigma +(-Q/RT)\\ lnA\;+\;nln[sinh(\alpha \sigma )]\;-\;Q/RT \end{array}\right.$$

where $n_{1}\;=\;\partial \ln\dot{\varepsilon }/\partial \ln\sigma$ and $\beta \;=\;\partial \ln\dot{\varepsilon }/\partial \sigma$. To determine the material constants in the constitutive equations, the peak stresses obtained under different deformation conditions were substituted into Equation (2). Based on the linear fitting results shown in Figure 4, the values of *n*_1_ and β in the α + β phase region and β single-phase region were calculated from the slopes of the $\ln\sigma \;-\;\ln\dot{\varepsilon }$ and $\sigma \;-\;\ln\dot{\varepsilon }$ plots, respectively. Subsequently, the material parameter α was obtained according to the relationship $\alpha \;=\;\beta /n_{1}$. The calculated α values for the lamellar Ti65 alloy were 0.0102 and 0.0217 MPa^−1^ in the α + β phase region and β single-phase region, respectively.

For a given temperature interval, the apparent activation energy for hot deformation, *Q*, is usually assumed to remain constant. On this basis, the Arrhenius-type equation can be further transformed, and the value of *Q* can be evaluated from the following relationship:

(3) $$Q\;=\;Rnk\;=\;R\;\cdot \;{\frac{\partial ln\dot{\varepsilon }}{\partial ln[sinh(\alpha \sigma )]}|}_{T}\;\cdot \;{\frac{\partial lnsinh(\alpha \sigma_{p})}{\partial (1/T)}|}_{\dot{\varepsilon }}$$

**Figure 5:**
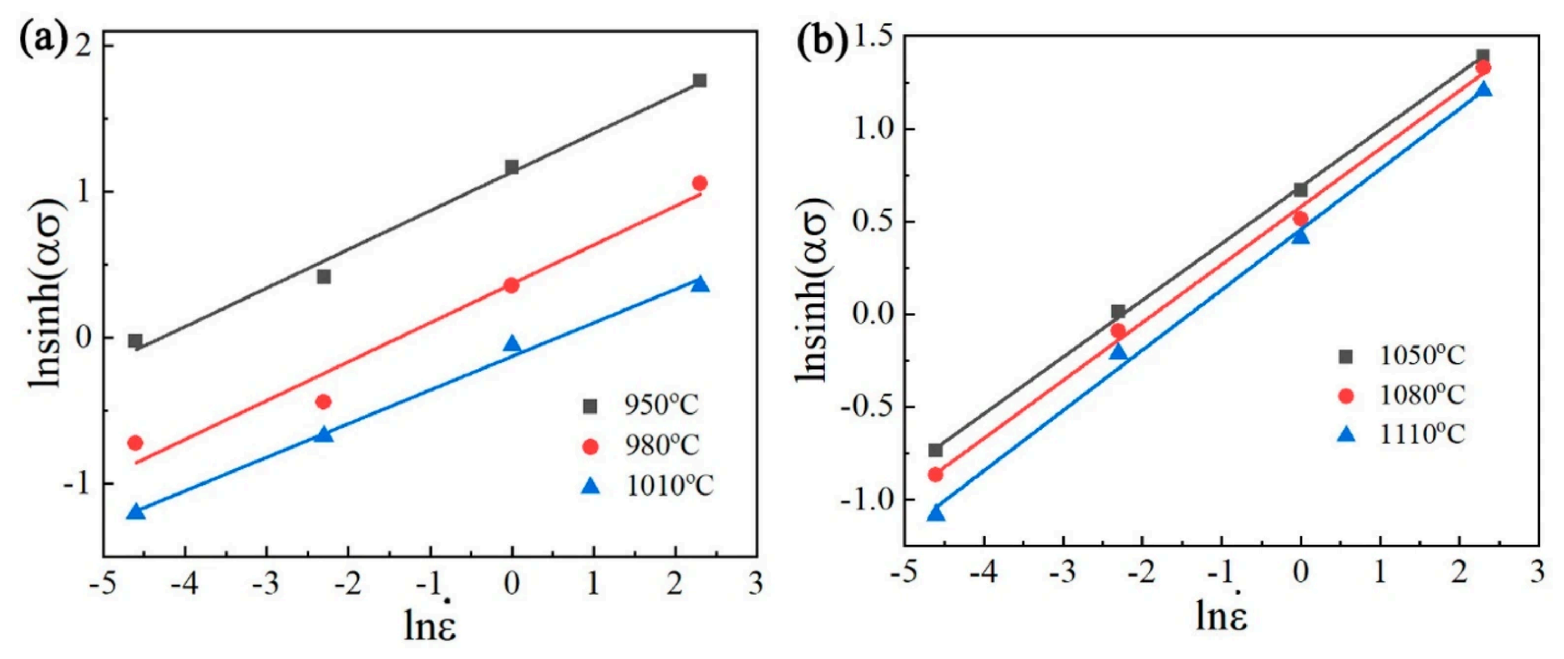
The correlation curves between ln[sinh(*ασ*)] and $\ln\dot{\varepsilon }$ in different phase regions of lamellar microstructure: (**a**) α + β phase field, (**b**) single β phase field.

**Figure 6:**
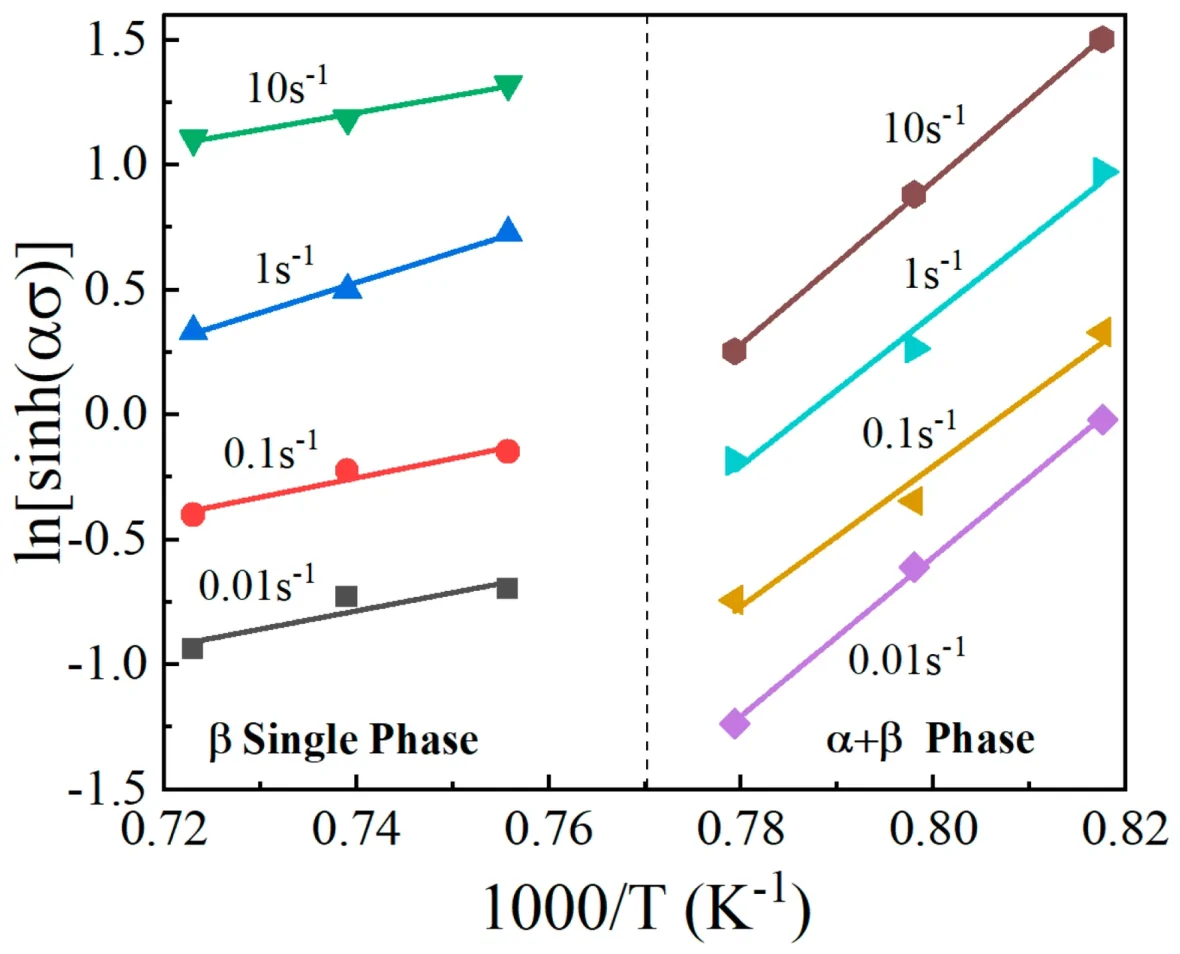
The correlation curves between ln[sinh(*ασ*)] and 1000/*T* in different phase regions of lamellar microstructure [5].

Based on Equation (3), the parameter related to strain-rate sensitivity can be obtained from the slope of the $\ln\left[\sinh\left(\alpha \sigma \right)\right]\;-\;\ln\dot{\varepsilon }$ plots, and the corresponding linear fitting results are shown in Figure 5. In a similar manner, the temperature-dependent term in Equation (3) can be calculated from the slope of the $\ln\left[\sinh\left(\alpha \sigma \right)\right]\;-\;(1/T)$ plots. The detailed fitting relationships are presented in Figure 6. For improved readability, 1000/T, rather than 1/T, was used as the abscissa in Figure 6. Therefore, the slope obtained directly from Figure 6 is $K\;=\;\partial ln[\sinh\left(\alpha \sigma \right)]/\partial (1000/T)$. Since $1/T\;=\;\left(1000/T\right)/1000$, a factor of 1000 must be included when calculating the apparent activation energy, i.e., Q = 1000RnK.

The calculated apparent activation energies based on the peak-stress data are 1050.27 kJ/mol in the α + β phase region and 203.51 kJ/mol in the β single-phase region. It should be emphasized that the activation energy obtained from the Arrhenius-type constitutive relationship is an apparent, phenomenological parameter reflecting the overall temperature sensitivity of the flow stress rather than the energy barrier of a single atomic diffusion mechanism. It is generally accepted that the self-diffusion activation energies of pure α-Ti and β-Ti are approximately 204 and 153 kJ/mol, respectively [34]. The activation energy obtained in the α + β phase region is therefore much higher than the self-diffusion activation energies of both α-Ti and β-Ti. In the β single-phase region, the obtained value is relatively close to the reported diffusion-related activation energies of β-Ti, supporting the predominance of diffusion-assisted dislocation recovery. In contrast, the markedly higher value in the α + β region results from the coupled contribution of several strongly temperature-dependent processes. With increasing temperature toward the β-transus, the phase fraction changes rapidly, while dislocation rearrangement, α-lamella kinking and fragmentation, α/β interfacial migration, dynamic spheroidization and α → β transformation occur concurrently. Consequently, the strong temperature sensitivity associated with these coupled microstructural processes is incorporated into the fitted apparent activation energy, giving rise to a value substantially higher than the self-diffusion activation energy of pure Ti. Similarly high apparent activation energies have been reported for other titanium alloys with lamellar microstructures during deformation in the α + β region [35,36,37]. The apparent activation energy calculated in the β single-phase region is only slightly higher than the self-diffusion activation energy of β-Ti. This suggests that dynamic recovery is likely to be the dominant restoration mechanism during deformation in the β region. Meanwhile, limited dynamic recrystallization of the β phase may also occur under certain deformation conditions. This interpretation is consistent with the evolution tendency of the flow stress curves, in which the β region deformation exhibits a relatively weaker flow-softening behavior compared with that in the α + β phase region.

It should be noted that the apparent activation energy obtained from the Arrhenius model should not be regarded as an intrinsic and invariant material constant. Within each phase region, a single effective activation energy is used at a given strain level to represent the overall temperature dependence of the flow stress. This treatment is a phenomenological approximation. In particular, in the α + β region, the relative fractions of α and β phases vary with temperature, while the contributions of α-lamella fragmentation, dynamic spheroidization, interfacial migration, dynamic recovery, and α → β transformation also evolve. Therefore, the calculated activation energy represents an effective value averaged over the investigated temperature interval rather than the activation barrier of a single microscopic process. In the β region, the assumption of a single effective activation energy is comparatively more reasonable because deformation occurs predominantly in the β phase, although temperature-dependent recovery and recrystallization processes may still contribute.

To improve the accuracy of flow-stress prediction, the influence of deformation temperature on the strain-rate-dependent deformation response was further considered. Accordingly, the Zener–Hollomon parameter was introduced to incorporate the combined effects of temperature and strain rate into the constitutive description. The expression of *Z* is given as:

(4) $$Z\;=\;\dot{\varepsilon }\exp\left(\frac{Q}{RT}\right)\;=\;A{\left[sinh(\alpha \sigma )\right]}^{n}$$

**Figure 7:**
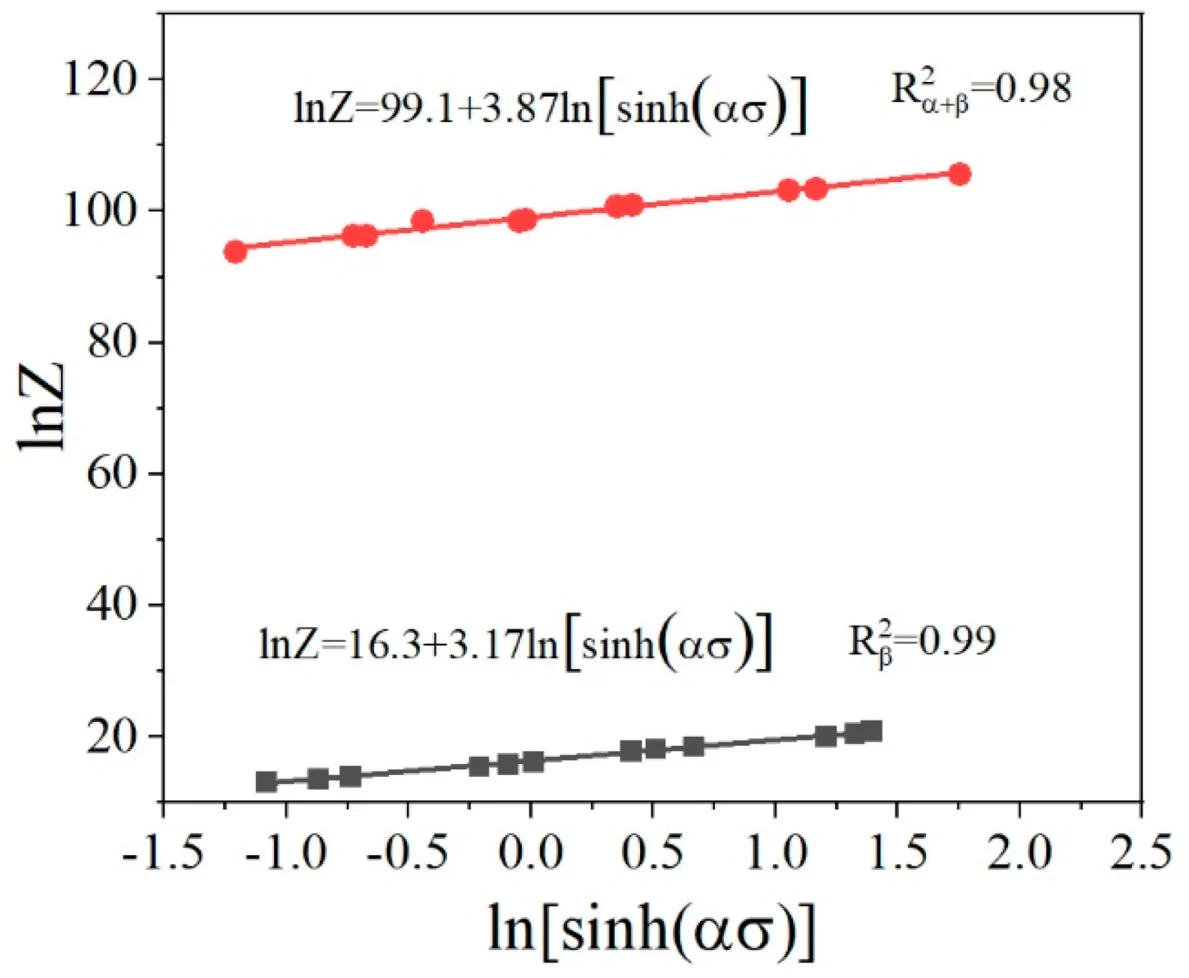
The correlation curves between ln*Z*-ln[sinh(*ασ*)] in different phase regions of lamellar microstructure.

After taking the natural logarithm of both sides of Equation (3), a linear relationship between ln Z and ln[sinh(ασ)] can be obtained, as expressed in Equation (5). Accordingly, ln A is evaluated from the intercept of the linear fitting curve in Figure 7.

(5) lnZ = lnA + nln[sinh(ασ)]

According to the material parameters determined above, the Arrhenius-type constitutive models for the Ti65 alloy in the α + β phase region and β single-phase region were established separately. The final constitutive equations are expressed as follows:

(6) $$\alpha \;+\;\beta \;\mathrm{phase}\;\mathrm{region}:\;\dot{\varepsilon }\;=\;1.04\;\times \;10^{43}{\left[sinh(0.0102\sigma )\right]}^{3.944}exp(-\frac{1,050,269}{RT})$$

(7) $$\beta \;\mathrm{phase}\;\mathrm{region}:\;\dot{\varepsilon }\;=\;1.17\;\times \;10^{7}{\left[sinh(0.0217\sigma )\right]}^{3.181}exp(-\frac{203,506}{RT})$$

However, the conventional Arrhenius-type equation in Equation (1) does not explicitly account for the influence of strain on flow stress during hot deformation. Previous studies have shown that strain can markedly affect the material constants involved in the constitutive model [38,39]. Therefore, to improve the prediction accuracy of the flow stress of the Ti65 alloy, it is necessary to establish the strain-dependent relationships of the material parameters in the constitutive equation. These relationships can be expressed in matrix form, as shown in Equation (8).

(8) $$\left[\begin{array}{c} ln\;A\\ \alpha \\ n\\ Q \end{array}\right]\;=\;\left[\begin{array}{ccc} B_{0} & \ldots & B_{n}\\ C_{0} & \ldots & C_{n}\\ D_{0} & \ldots & D_{n}\\ E_{0} & \ldots & E_{n} \end{array}\right]\left[\begin{array}{c} \varepsilon^{0}\\ \varepsilon^{1}\\ \vdots \\ \varepsilon^{n} \end{array}\right]$$

After incorporating both the Zener–Hollomon parameter and the strain effect, the flow stress can be calculated and predicted using Equation (9). To assess the influence of polynomial order and minimize the risk of overfitting, second- to sixth-order polynomial functions were systematically evaluated for describing the strain dependence of lnA, *n*, *Q*, and *α*. The fitting quality was quantified using the coefficient of determination (*R*^2^), adjusted coefficient of determination ($R_{adj}^{2}$), and root-mean-square error (RMSE). The corresponding statistical results are summarized in Table S1. With increasing polynomial order, the fitting accuracy generally improved, and the sixth-order polynomial provided the highest adjusted *R*^2^ and the lowest RMSE for all four material parameters in both phase regions. To further examine the possibility of overfitting, a leave-one-condition-out cross-validation was performed at the constitutive-model level. In each iteration, one complete temperature–strain-rate condition was excluded from model calibration and subsequently predicted using the model established from the remaining deformation conditions. The results are shown in Table S2. No deterioration in cross-validation performance was observed for the sixth-order polynomial compared with the lower-order functions. Therefore, the sixth-order polynomial was retained as a common-order representation of the strain-dependent material parameters. It should be noted that the polynomial functions are intended only for interpolation within the experimentally investigated strain range of 0.10–0.90 and should not be extrapolated beyond this range.

**Figure 8:**
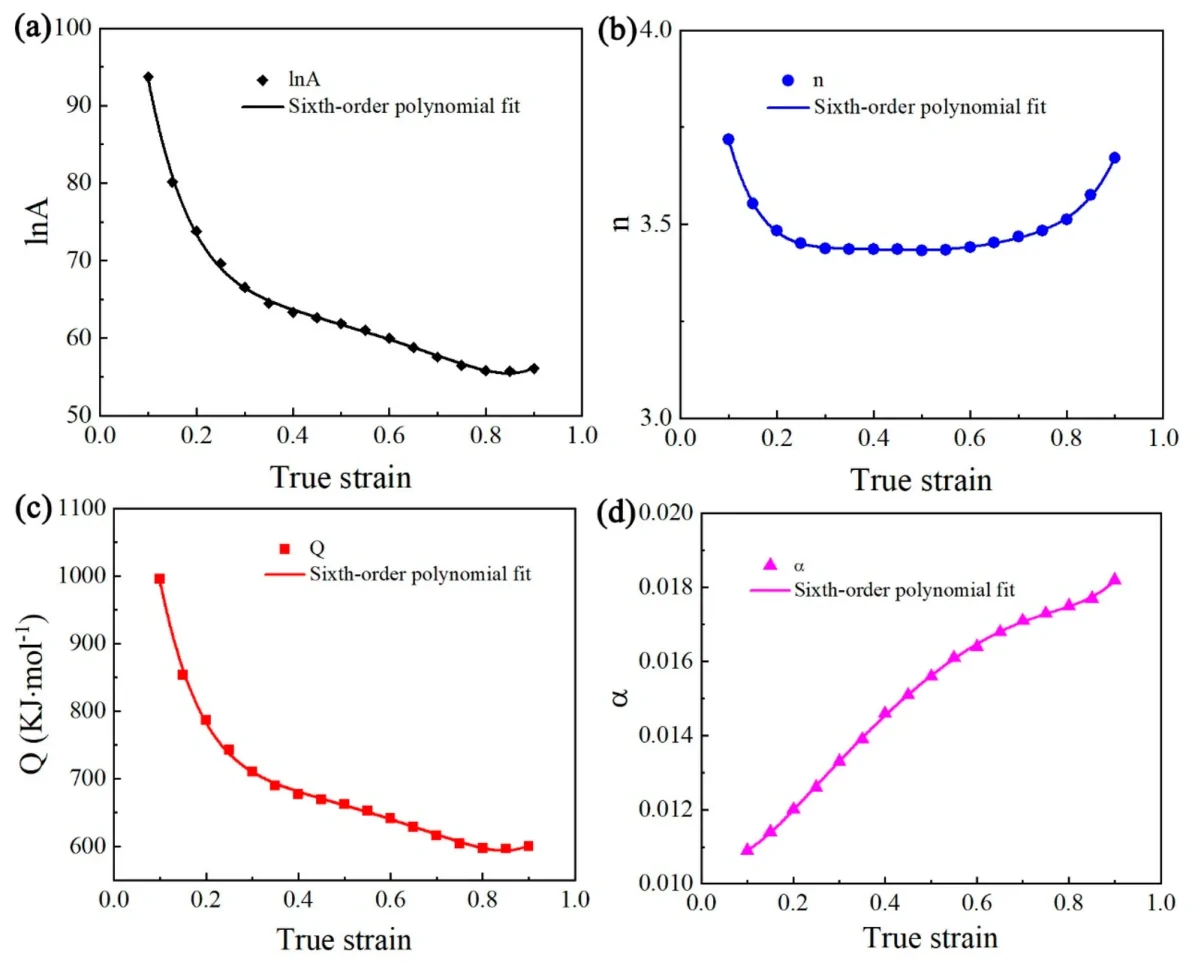
Correlation fitting curves between four material parameters ((**a**) ln*A*, (**b**) *n*, (**c**) *Q*, (**d**) *α*) and strain value by employing the sixth-order polynomial fitting method in the α + β phase field.

**Figure 9:**
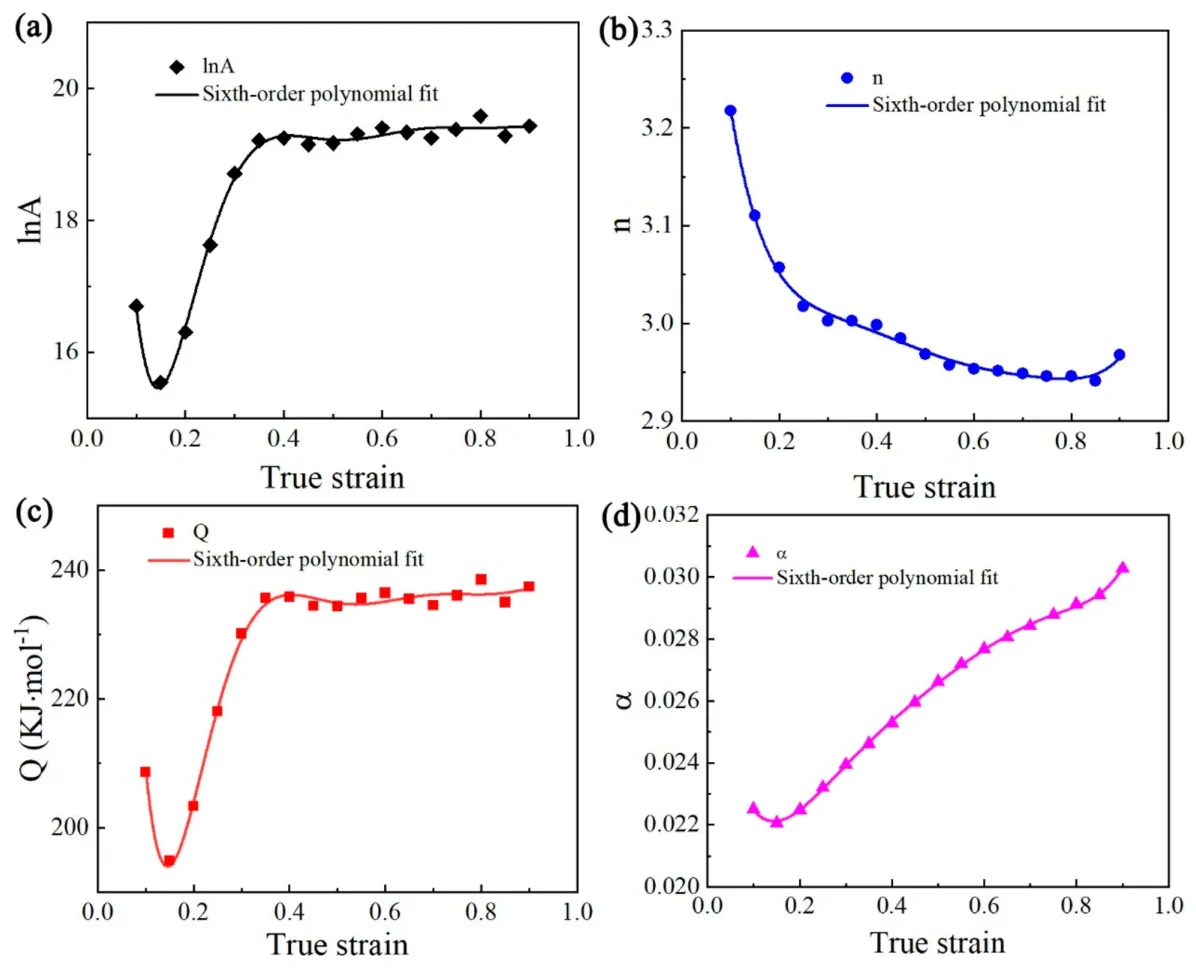
Correlation fitting curves between four material parameters ((**a**) ln*A*, (**b**) *n*, (**c**) *Q*, (**d**) *α*) and strain value by employing the sixth-order polynomial fitting method in the single β phase field.

The corresponding relationships between the material constants and strain for the lamellar microstructure in different phase regions are presented in Figure 8 and Figure 9. The results indicate that the material parameters vary noticeably with increasing strain. For example, the apparent activation energy of the lamellar Ti65 alloy fluctuates between 597 and 1050 kJ/mol in the α + β phase region, whereas it varies within a narrower range of 195–238 kJ/mol in the β single-phase region. The polynomial coefficients and the corresponding functional expressions describing the strain-dependent material parameters in the constitutive equations are summarized in Table 2 and Table 3.

(9) $$\sigma \;=\;\frac{1}{\alpha }ln\left\{{\left(\frac{Z}{A}\right)}^{\frac{1}{n}}\;+\;{\left[{\left(\frac{Z}{A}\right)}^{\frac{2}{n}}\;+\;1\right]}^{\frac{1}{2}}\right\}$$

**Figure 10:**
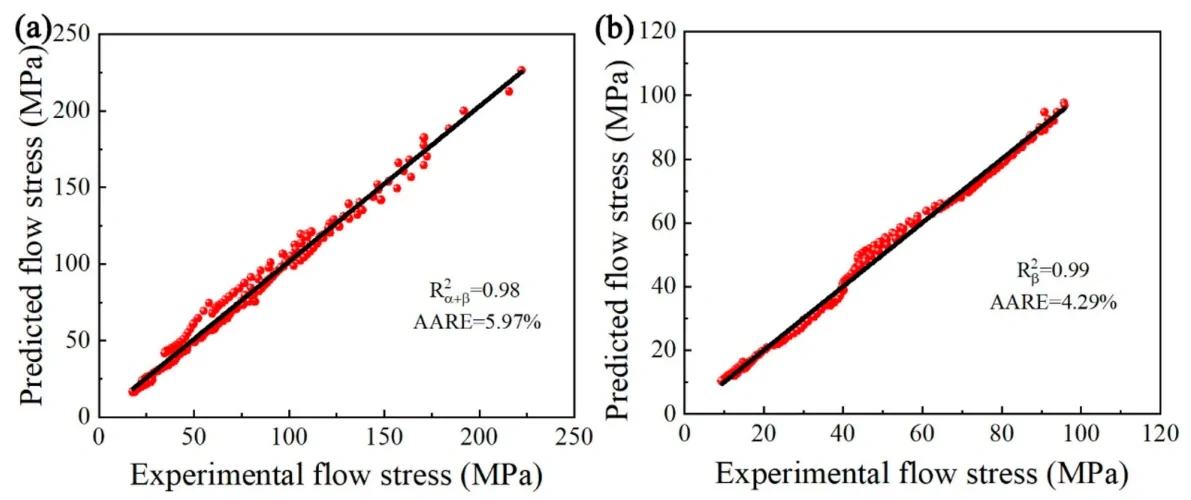
The relevance of the correlation between the experimental flow stress and the estimated flow stress of lamellar microstructure with different phase fields using the Arrhenius-type constitutive model: (**a**) α + β phase region, (**b**) single β phase region.

Figure 10 compares the agreement between the calculated and experimental flow stresses of the lamellar Ti65 alloy in different phase regions obtained using the Arrhenius-type constitutive model. To quantitatively evaluate the fitting accuracy of the established model, the correlation coefficient R and the average absolute relative error AARE were employed. These two statistical indicators are commonly used to assess the agreement between experimental and calculated values. The corresponding expressions are given as follows:

(10) $$R\;=\;\frac{\sum_{i=1}^{N}\left(X_{i}\;-\;\bar{X})(Y_{i}\;-\;\bar{Y}\right)}{\sqrt{\sum_{i=1}^{N}{\left(X_{i}\;-\;\bar{X}\right)}^{2}\sum_{i=1}^{N}{\left(Y_{i}\;-\;\bar{Y}\right)}^{2}}}$$

(11) $$AARE\;=\;\frac{1}{N}\sum_{i=1}^{N}\left|\frac{Y_{i}\;-\;X_{i}}{X_{i}}\right|\;\times \;100\%$$

where $X_{i}$ and $Y_{i}$ represent the experimental and predicted flow stress values, respectively; $\bar{X}$ and $\bar{Y}$ are the average values of the experimental and predicted flow stresses, respectively; and *N* is the total number of data points used for model evaluation.

The established models exhibit good fitting accuracy and agreement with the experimental flow stresses for the lamellar Ti65 alloy in both phase regions. In the α + β phase region, the correlation coefficient R and average absolute relative error AARE are 0.98 and 5.97%, respectively. In the β single-phase region, the corresponding values are 0.99 and 4.29%, respectively. These results indicate that the established strain-compensated Arrhenius-type constitutive equation can accurately describe the hot deformation behavior of the lamellar Ti65 alloy. Moreover, the slightly higher R value and lower AARE value in the β single-phase region suggest that the model exhibits better predictive capability in the β region than in the α + β phase region. The cross-validation results shown in Table S2 further demonstrate satisfactory generalization capability of the constitutive model.

### 3.3. Hot Processing Map

At present, two main types of processing maps have been reported for hot deformation analysis: the Raj processing map based on an atomistic model [40] and the processing map established using the dynamic materials model (DMM) [41]. In general, the Raj map is mainly applicable to pure metals or relatively simple alloys, and the determination of its model parameters usually requires extensive theoretical calculations. Therefore, this approach has certain limitations and is not suitable for constructing the processing map of the multi-component Ti65 alloy.

In contrast, the DMM-based processing map has been widely used to evaluate the hot workability of various metallic materials, including aluminum alloys [42], titanium alloys [24] and high-strength steels [43]. According to the DMM proposed by Prasad et al., the workpiece undergoing hot deformation can be regarded as a non-linear energy-dissipation system [44]. The flow stress is assumed to follow a power-law relationship with strain rate, and the strain-rate sensitivity index can be used to calculate the power dissipation efficiency and identify flow-instability domains.

During hot deformation, the total external power input (P) is generally partitioned into two complementary parts: the power content (G), which is consumed by plastic deformation, and the power co-content (J), which is dissipated through microstructural evolution. Most of the energy associated with plastic deformation is converted into heat, while only a small fraction is stored in the material in the form of crystal defects. By contrast, the energy dissipated through microstructural evolution is closely related to deformation-induced metallurgical processes, such as dynamic recovery, dynamic recrystallization, dynamic phase transformation and globularization of lamellar microstructures. Accordingly, the total power input can be expressed as follows:

(12) $$P\;=\;\sigma \dot{\varepsilon }\;=\;G\;+\;J\;=\;\int_{0}^{\dot{\varepsilon }}\sigma d\dot{\varepsilon }\;+\;\int_{0}^{\sigma }\dot{\varepsilon }d\sigma$$

In the above equation, σ and $\dot{\varepsilon }$ denote the flow stress and strain rate during hot deformation, respectively. *G* is defined as the dissipator content, while *J* represents the dissipator co-content. For a fixed strain, the dependence of true stress on strain rate can be described by the following relationship:

(13) $$ln\sigma \;=\;a_{1}\;+\;a_{2}ln\dot{\varepsilon }\;+\;a_{3}{\left(ln\dot{\varepsilon }\right)}^{2}\;+\;a_{4}{\left(ln\dot{\varepsilon }\right)}^{3}$$

where $a_{1}$–$a_{4}$ are material constants determined by fitting the experimental data. The dissipator co-content *J* can then be expressed as follows:

(14) $$J\;=\;\int_{0}^{\sigma }\dot{\varepsilon }d\sigma \;=\;\frac{m}{m\;+\;1}\sigma \dot{\varepsilon }$$

For an ideal linear dissipative system, the dissipator co-content reaches its maximum value, *J*_max_, when the strain-rate sensitivity exponent *m* is equal to 1. In the dynamic materials model, the power dissipation efficiency *η* is commonly used to evaluate the hot workability of metallic materials. This parameter represents the fraction of the total power input that is dissipated through microstructural evolution during hot deformation. It can be calculated using the following equation:

(15) $$\eta \;=\;\frac{J}{J_{max}}\;=\;\frac{2m}{m\;+\;1}$$

To delineate the unsafe processing domains, the flow-instability criterion proposed by Prasad et al. was adopted [45]. This criterion was developed from the extremum principle of irreversible thermodynamics for continuum systems, and the instability condition is given by:

(16) $$\frac{dD}{d\dot{\varepsilon }}\;<\;\frac{D}{\dot{\varepsilon }}$$

where *D* represents the dissipation function. Since the energy consumed by microstructural evolution during hot deformation is associated with the dissipator power co-content, *D* can be replaced by *J* according to the dynamic materials model, namely:

(17) $$\frac{dJ}{d\dot{\varepsilon }}\;<\;\frac{J}{\dot{\varepsilon }}$$

(18) $$\frac{\partial lnJ}{\partial ln\dot{\varepsilon }}\;\leq \;1$$

According to the maximum entropy production rate principle, the flow-instability criterion used in the processing map is finally obtained as:

(19) $$\xi (\dot{\varepsilon })\;=\;\frac{\partial ln(\frac{m}{m\;+\;1})}{\partial ln\dot{\varepsilon }}\;+\;m\;<\;0$$

The processing map was constructed by superimposing the power dissipation map and the flow-instability map. At a given strain, the power dissipation efficiency *η* was calculated under different combinations of deformation temperature and strain rate, and the obtained *η* values were plotted as contour lines to generate the power dissipation map. Physically, *η* represents the relative entropy-production rate associated with microstructural evolution during hot deformation. Similarly, the instability map was established based on the flow-instability criterion and was used to identify unstable deformation domains in the two-dimensional space of deformation temperature and strain rate.

**Figure 11:**
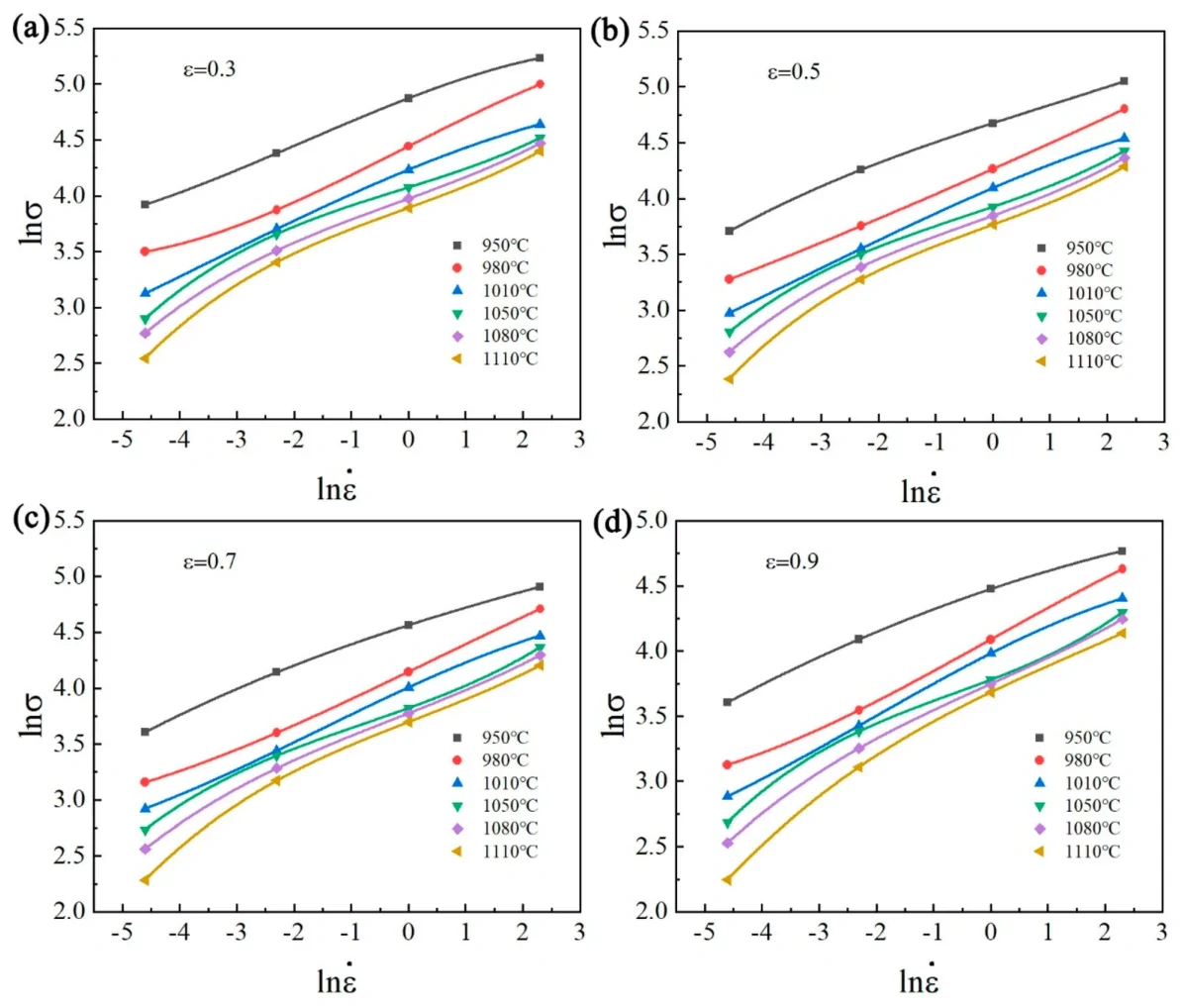
The correlation curves between ln*σ* and $\ln\dot{\varepsilon }$ of lamellar microstructure under different deformation conditions of the Ti65 alloy: (**a**) *ε* = 0.3, (**b**) *ε* = 0.5, (**c**) *ε* = 0.7, (**d**) *ε* = 0.9.

To calculate the power dissipation efficiency, the strain-rate sensitivity exponent *m* was first determined from the relationship between lnσ and $\ln\dot{\varepsilon }$. The corresponding fitting results at different deformation temperatures are shown in Figure 11. It can be observed that the strain rate has a pronounced influence on the flow stress, and the stress increases markedly with increasing strain rate. In addition, the effect of temperature on flow stress differs between the α + β phase region and the β single-phase region. In the lower-temperature α + β phase region, the flow stress is strongly affected by deformation temperature. By contrast, in the β single-phase region, the temperature sensitivity of flow stress becomes weaker. This tendency is particularly evident at high strain rates, where the variation in flow stress with increasing temperature is relatively limited.

**Figure 12:**
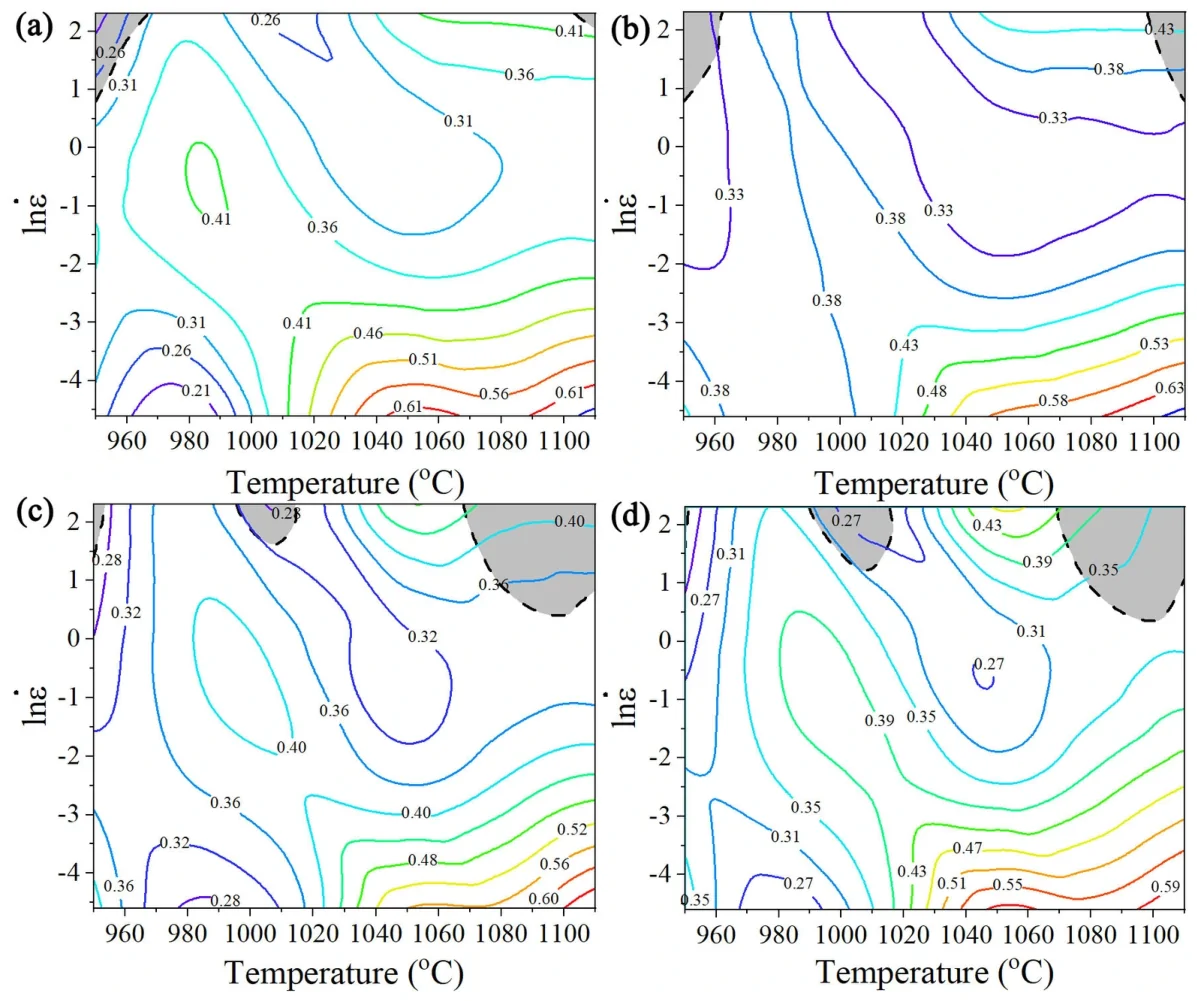
Hot processing map of lamellar microstructure with different strains: (**a**) *ε* = 0.3, (**b**) *ε* = 0.5, (**c**) *ε* = 0.7, (**d**) *ε* = 0.9.

The instability parameter in the processing map was calculated according to Equation (19). Specifically, the relationship between $\ln\left(m/m\;+\;1\right)$ and $\ln\dot{\varepsilon }$ was fitted using a polynomial function, and the corresponding slope under each deformation condition was then obtained. By adding this slope to the strain-rate sensitivity exponent *m*, the instability parameter $\xi (\dot{\varepsilon })$ was determined. The final processing map was constructed by superimposing the power dissipation map and the flow-instability map. Since the flow stress of Ti65 alloy evolves continuously with strain, the strain-rate sensitivity *m*, power dissipation efficiency *η*, and flow-instability parameter ξ are also strain-dependent. Therefore, processing maps were additionally constructed at true strains of 0.3, 0.5, 0.7, and 0.9 to evaluate the evolution of hot workability with increasing deformation. The corresponding processing maps are shown in Figure 12. It indicates that, at relatively low strains (ε<0.5), the instability domains are mainly located in the low-temperature/high-strain-rate region. The total area occupied by the instability domains is relatively limited, suggesting that the alloy exhibits good hot workability at low strains and is less susceptible to flow instability during hot deformation. With increasing strain, the processing-map characteristics change progressively. When the strain exceeds 0.7, an additional instability domain emerges in the intermediate-temperature/high-strain-rate region, approximately within 995–1015 °C and 5–10 s^−1^. As the strain further increases to 0.9, this intermediate-temperature instability domain expands slightly. These results demonstrate that the hot processing window of the lamellar Ti65 alloy is strain-dependent, and the tendency toward flow instability becomes more pronounced at larger strains.

**Figure 13:**
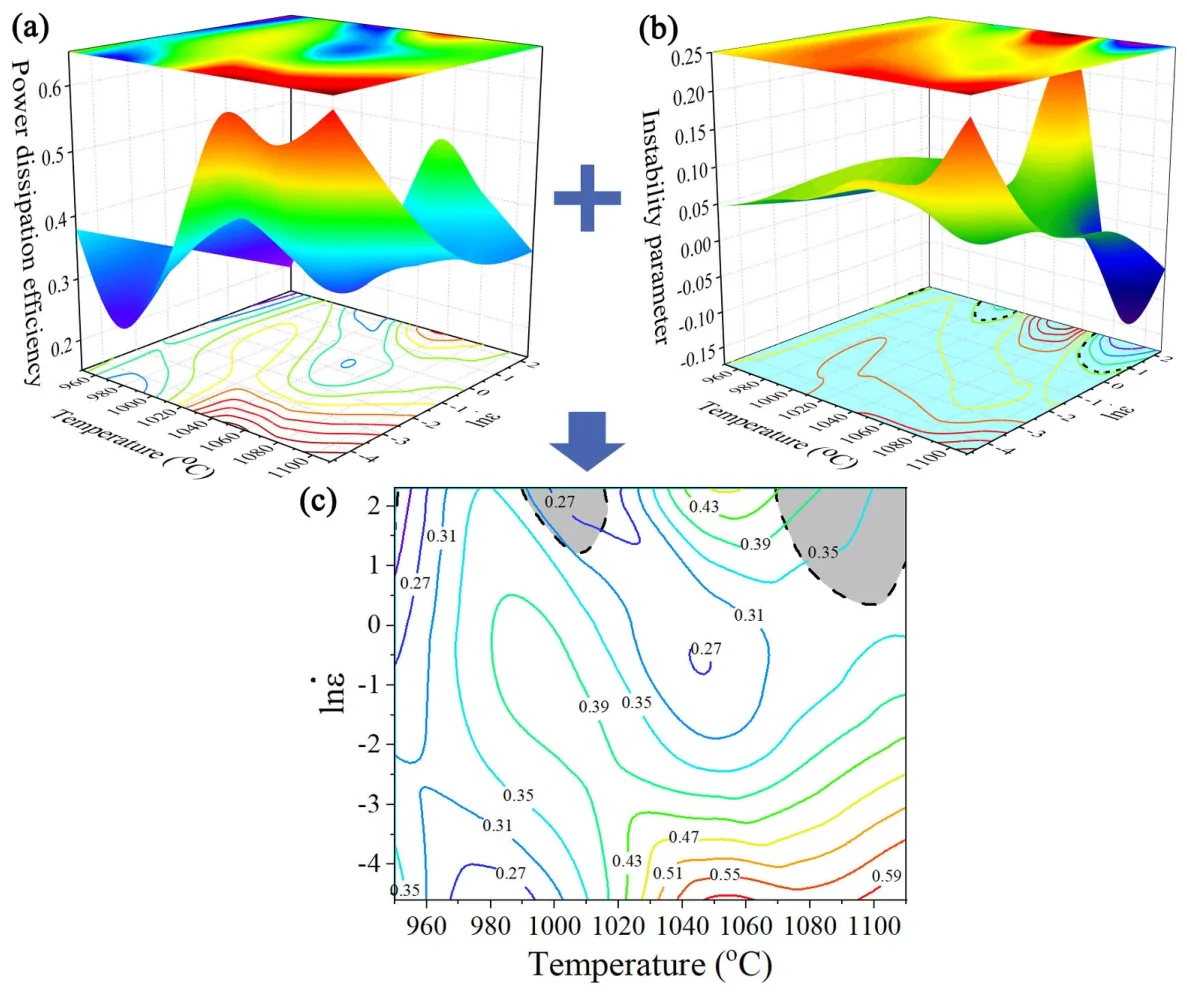
(**a**) Power dissipation efficiency map of lamellar microstructure under the strain of 0.9, (**b**) plastic flow-instability map, (**c**) hot processing map.

Figure 13 shows the processing map of the lamellar Ti65 alloy at a strain of 0.9. The processing map established based on the dynamic materials model can be used to predict suitable hot-working domains and avoid the formation of microstructural defects [46]. Su et al. [25] also employed processing maps to identify the stable and unstable deformation regions of the short-term high-temperature titanium alloy DsTi700. Their results showed that obvious flow localization occurred in the instability domain at high strain rates, indicating an unstable microstructural state.

As shown in Figure 13c, two instability domains can be identified during hot deformation of the lamellar Ti65 alloy. One is located above the phase-transformation temperature at high strain rates, corresponding to 1060–1110 °C and 1.65–10 s^−1^. The other appears in the upper part of the α + β phase region below the phase-transformation temperature, within the range of 990–1020 °C and 3.5–10 s^−1^. When deformation is conducted within these domains, the alloy is more likely to undergo flow instability. Typical plastic instability modes in titanium alloys include adiabatic shear bands, localized flow and wedge cracking [4]. Macroscopic instability or cracking after hot deformation can usually be identified from the external appearance of the compressed specimens, whereas internal microstructural instability must be further confirmed by microstructural characterization. Therefore, although the processing map provides an effective basis for optimizing the hot-working window and improving the overall mechanical performance of the alloy, possible defect formation during actual deformation cannot be completely excluded. A combined analysis of processing maps and microstructural evolution is therefore necessary to further suppress plastic instability during hot working [47].

**Figure 14:**
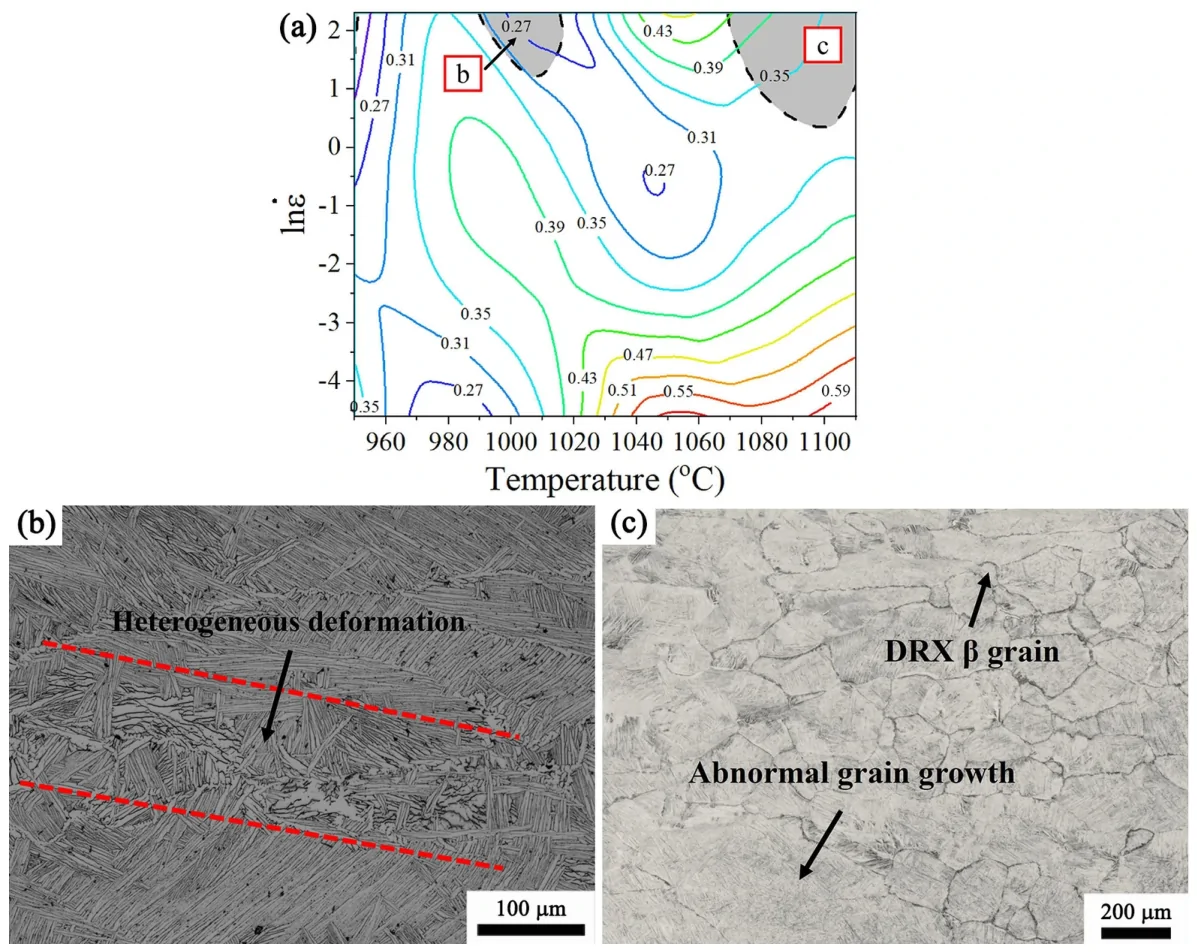
Hot processing map and the microstructure characterization of the corresponding instable region: (**a**) hot processing map under the strain of 0.9, (**b**) microstructure at medium temperature and high strain rate, 1010 °C, 10 s^−1^, (**c**) microstructure at high temperature and high strain rate, 1110 °C, 10 s^−1^.

Figure 14 shows the processing map of the lamellar Ti65 alloy at a strain of 0.9 and the microstructures corresponding to the instability domains. As shown in Figure 14a, two instability regions, marked as regions b and c, can be identified. Their representative microstructures are presented in Figure 14b,c, respectively. At 1010 °C and 10 s^−1^, the alloy was deformed in the upper α + β phase region, where partial α → β transformation may occur. Under the combined effects of large strain, high strain rate and adiabatic temperature rise, localized plastic deformation was promoted, resulting in non-uniform spheroidization of lamellar α. As indicated by the red dashed lines in Figure 14b, a band-like region with heterogeneous α spheroidization was formed, suggesting a local instability feature that may cause stress concentration. Figure 14c shows the microstructure deformed in the β single-phase region at 1110 °C and 10 s^−1^. At this condition, the deformation condition lies within the instability domain predicted by the DMM instability criterion (ξ < 0). The corresponding microstructure exhibits pronounced β-grain coarsening. However, grain coarsening itself should not be regarded as direct evidence of flow instability, because the high deformation temperature can also enhance β-grain-boundary mobility and promote grain growth. Therefore, the coarse β-grain morphology observed under this condition is interpreted as a microstructural feature accompanying the high-temperature/high-strain-rate instability domain, whereas the classification of flow instability is primarily based on the negative instability parameter obtained from the processing map. Although extensive dynamic recrystallization of β grains occurred, abnormal grain growth was also observed locally. This resulted in a mixed-grain structure composed of fine recrystallized grains and coarsened β grains. Such microstructural heterogeneity may induce non-uniform deformation during subsequent processing and negatively affect the mechanical properties of the final forged components.

**Figure 15:**
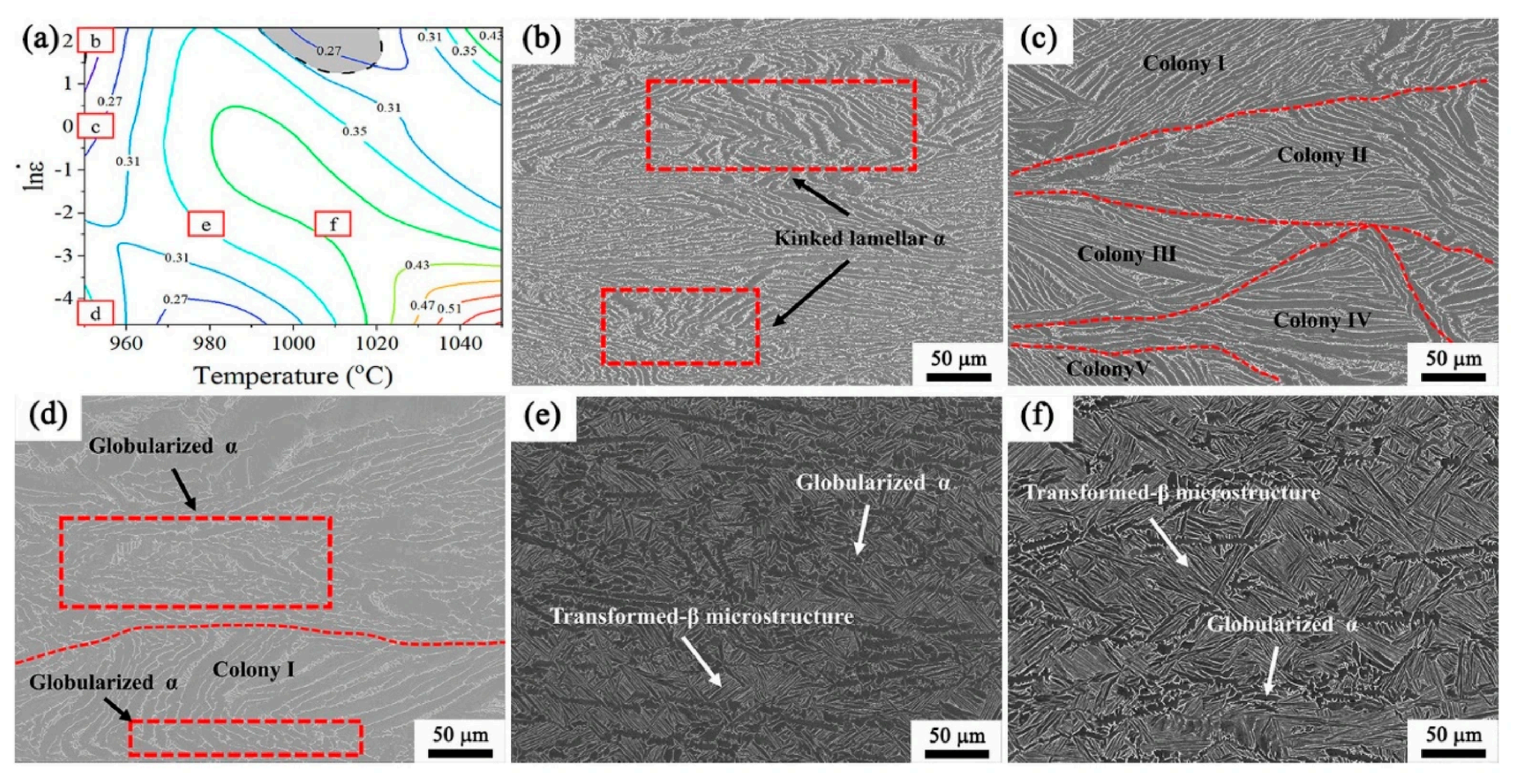
Hot processing map of lamellar microstructure and the microstructure characterization of the corresponding stable region in α + β phase region: (**a**) hot processing map under the strain of 0.9, (**b**–**f**) the corresponding microstructure deformed at different processing parameters: (**b**) 950 °C, 10 s^−1^, (**c**) 950 °C, 1 s^−1^, (**d**) 950 °C, 0.01 s^−1^, (**e**) 980 °C, 0.1 s^−1^, (**f**) 1010 °C, 0.1 s^−1.^

Figure 15a shows the processing map of the lamellar Ti65 alloy deformed in the α + β phase region. To correlate the processing-map characteristics with microstructure evolution, five representative specimens with different power dissipation efficiencies were selected for microstructural analysis, as marked by the red boxes b-f in Figure 15a. Regions b and c correspond to relatively low power dissipation efficiencies (*η* < 0.27). The corresponding microstructures after deformation at 950 °C/10 s^−1^ and 950 °C/1 s^−1^ are shown in Figure 15b,c, respectively. With increasing strain rate, the lamellar α phase changes from a colony-like arrangement, as indicated by colonies I–V in Figure 15c, to a more distorted morphology with local kinking and bending, as shown in Figure 15b. Regions d and e exhibit moderate power dissipation efficiencies (0.31 < *η* < 0.35). As shown in Figure 15d, partial spheroidization of lamellar α occurs after deformation at 950 °C and 0.01 s^−1^, but the spheroidization is highly heterogeneous and mainly limited to local regions within individual colonies. In colony I, most α lamellae still retain their plate-like morphology. This heterogeneous spheroidization may be related to the spatial arrangement and crystallographic orientation of α lamellae with respect to the loading direction. Previous studies have shown that “soft-oriented” α lamellae can activate multiple slip systems, such as basal and prismatic slip or prismatic and pyramidal <c + a> slip, thereby promoting a higher globularization efficiency. In Figure 15e, when the temperature increases to 980 °C at a strain rate of 0.1 s^−1^, the deformed microstructure consists of equiaxed α, elongated α and transformed β microstructure, including residual lamellar α and interlamellar β. Compared with the microstructure deformed at 950 °C, the degree of α spheroidization is further increased.

Region f in Figure 15a corresponds to a high-power dissipation efficiency range of (0.39 < *η* < 0.43), and the microstructure deformed at 1010 °C and 0.1 s^−1^ is shown in Figure 15f. Since this temperature is located in the upper α + β phase region and close to the β-rich region, a large amount of primary β phase participates in plastic deformation. As a result, a relatively high-volume fraction of transformed β microstructure is retained after deformation, as indicated by the white arrows in Figure 15f. Meanwhile, the elevated temperature also promotes partial spheroidization of the deformed lamellar α phase. Compared with Figure 15e, increasing the deformation temperature from 980 °C to 1010 °C at the same strain rate of 0.1 s^−1^ leads to a noticeable increase in the transformed β microstructure. However, the amount of lamellar α involved in deformation decreases at the higher temperature, resulting in a lower degree of α spheroidization. Overall, the microstructures obtained at 980 °C/0.1 s^−1^ and 1010 °C/0.1 s^−1^ both show a tendency to evolve toward a bimodal microstructure.

**Figure 16:**
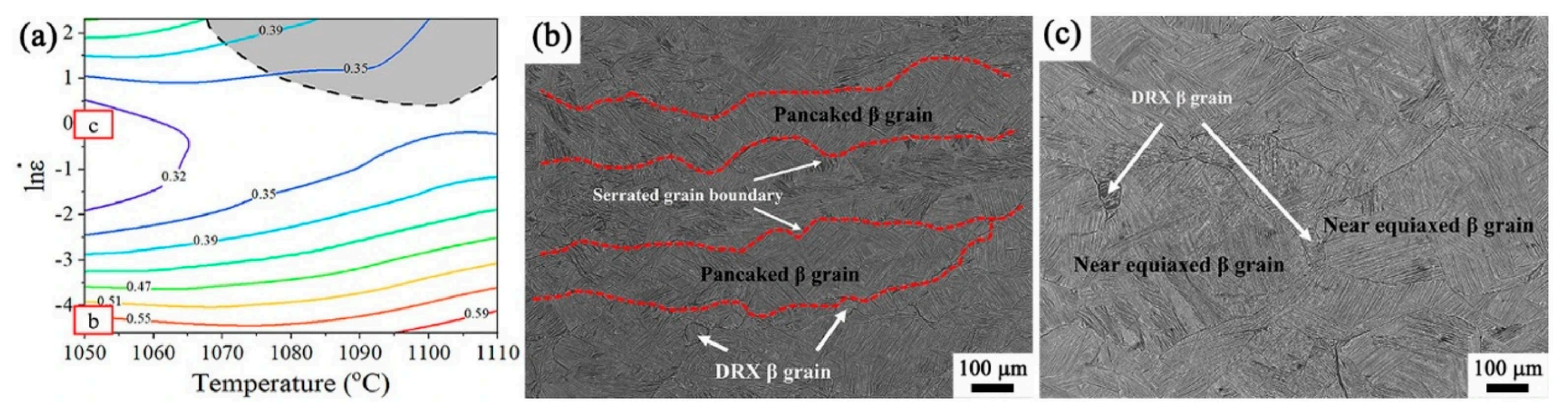
Hot processing map of lamellar microstructure and the microstructure characterization of the corresponding stable region in the single β phase region: (**a**) hot processing map under the strain of 0.9, (**b**,**c**) the corresponding microstructure deformed at different processing parameters: (**b**) 1050 °C, 0.01 s^−1^, (**c**) 1050 °C, 1 s^−1.^

Figure 16a shows the processing map of the lamellar Ti65 alloy deformed in the β single-phase region. To reveal the effect of power dissipation efficiency on microstructure evolution, two representative conditions, marked as b (1050 °C, 0.01 s^−1^) and c (1050 °C, 1 s^−1^), were selected for microstructural analysis. The corresponding microstructures are shown in Figure 16b,c, respectively. As the strain rate decreases and the *η* value increases, the β grains after deformation tend to be elongated along the material flow direction. Meanwhile, serrated grain boundaries and a small number of dynamically recrystallized β grains can be observed, indicating obvious migration of the prior β grain boundaries under deformation. These features may be responsible for the relatively high power dissipation efficiency under this condition. In contrast, after deformation at 1050 °C and 1 s^−1^, the prior β grains exhibit a higher degree of equiaxial morphology. A few dynamically recrystallized grains are also observed near the triple junctions, as indicated by the white arrows in Figure 16c. During subsequent cooling from the β single-phase region, continuous grain-boundary α and intragranular α_s_ precipitate from the deformed β matrix.

Based on the combined processing-map and microstructural analyses, the preferred hot-working conditions for the lamellar Ti65 alloy are located in the low-strain-rate region of the α + β phase field. Among the experimentally investigated conditions, deformation near 980 °C and 0.01–0.1 s^−1^ provides a favorable balance between flow stability and microstructural evolution. Under these conditions, the instability criterion is not satisfied, while α-lamella fragmentation and dynamic spheroidization are effectively promoted. At lower temperatures, spheroidization remains relatively heterogeneous, whereas increasing the temperature toward the β-transus increases the fraction of transformed β and reduces the amount of lamellar α participating in spheroidization. Therefore, a practical hot-working window centered around 980 °C and 0.01–0.1 s^−1^ is recommended for processing Ti65 alloy with an initial lamellar microstructure.

### 3.4. Microstructure Evolution Under Different Deformation Amounts

**Figure 17:**
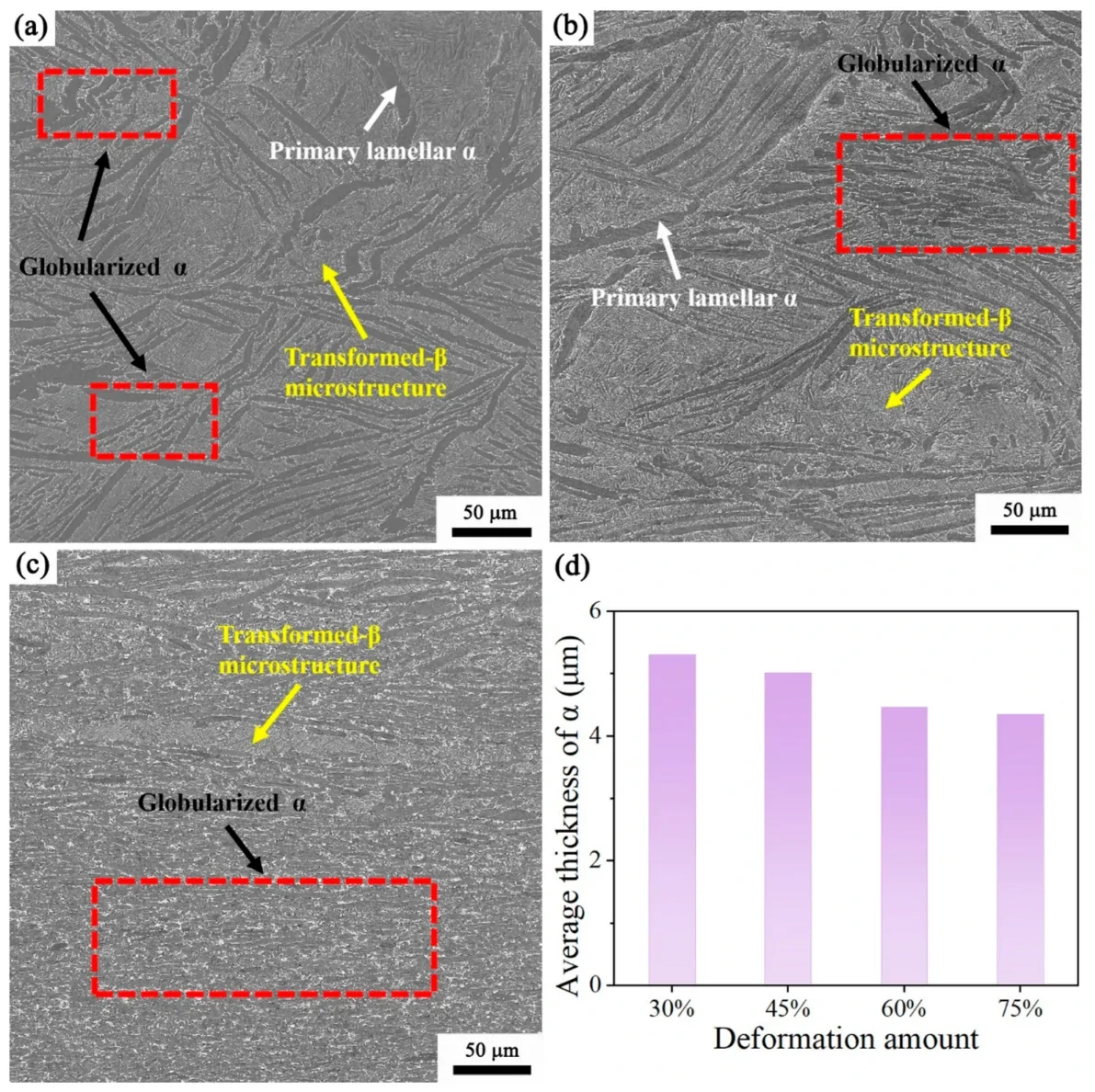
The microstructure and its quantitative statistics at parameters of 980 °C and 0.01 s^−1^ under different deformation amounts of lamellar microstructure: (**a**) 30%, (**b**) 45%, (**c**) 75%, (**d**) relationship between average thickness of primary lamellar α phase and deformation amounts.

The hot deformation behavior, constitutive modeling and processing-map construction of the lamellar Ti65 alloy have been discussed above. The results indicate that dynamic spheroidization of lamellar α is the dominant microstructural evolution mechanism in the α + β phase region. Since the local deformation amount is usually non-uniform during forging, it is necessary to further clarify the effect of strain on microstructure and crystallographic orientation during hot deformation. Figure 17 shows the microstructures of the lamellar Ti65 alloy deformed at 980 °C and 0.01 s^−1^ under different deformation amounts, together with the quantitative statistics of the average thickness of primary lamellar α. As shown in Figure 17a–c, the deformed microstructure is mainly composed of spheroidized equiaxed or nearly equiaxed α, residual elongated α with kinked or non-kinked morphology, short-rod-like α, and transformed β microstructure consisting of fine α_s_ lamellae and interlamellar β.

With increasing deformation amount, the fraction of transformed β microstructure gradually decreases, as marked by the yellow lines. When the deformation amount exceeds 60%, the transformed β fraction is reduced to below approximately 10%. This indicates that, during hot forging at 980 °C, the lamellar Ti65 alloy gradually evolves from a bimodal-like microstructure toward an equiaxed microstructure with increasing deformation amount. Meanwhile, the volume fraction of dynamically spheroidized α increases continuously, as indicated by the red boxes. The statistical results in Figure 17d further show that the average thickness of primary lamellar α decreases with increasing deformation amount. Therefore, a larger deformation amount is beneficial for promoting α-lamella fragmentation, dynamic spheroidization and microstructural refinement toward equiaxed grains.

**Figure 18:**
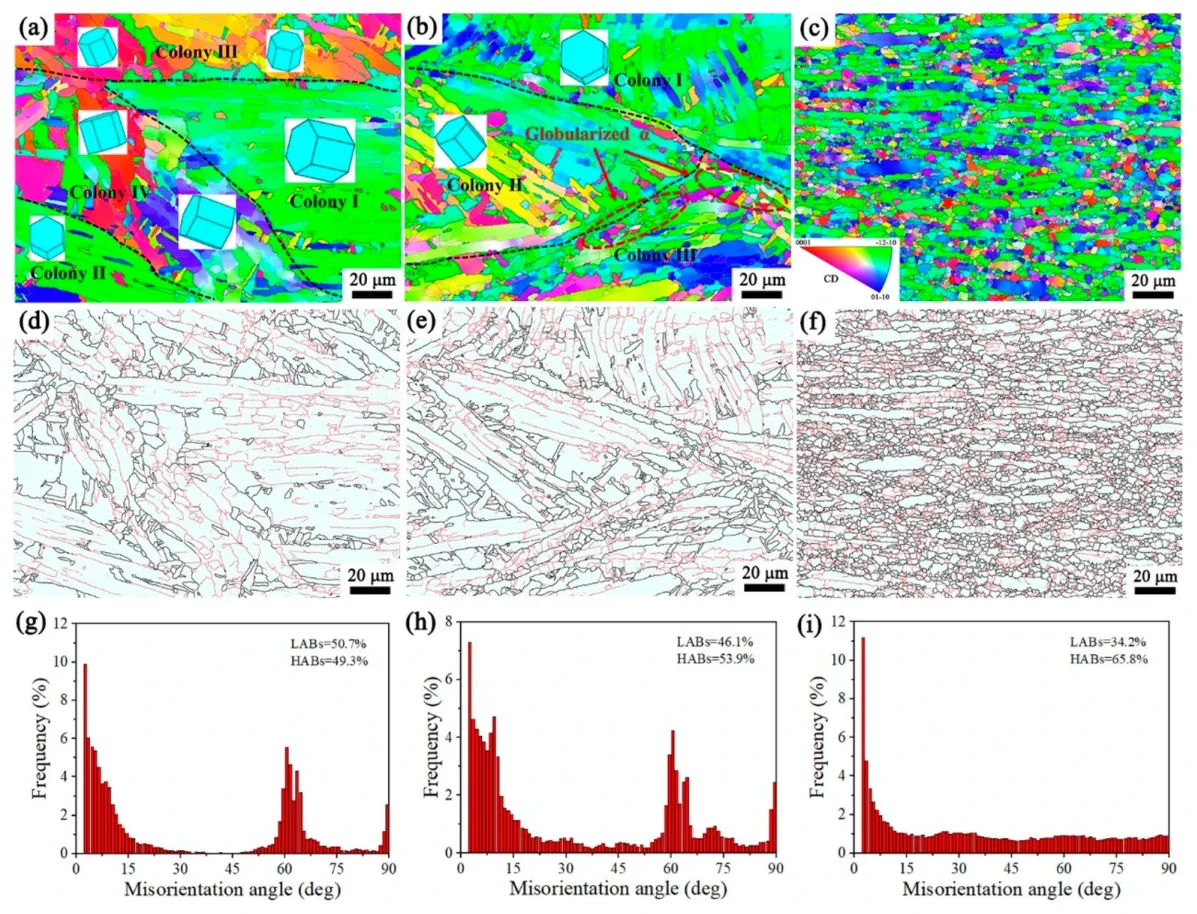
The microstructure under parameters of 980 °C and 0.01 s^−1^ with different deformation amounts: (**a**–**c**) IPF maps and the colors relative to CD, (**d**–**f**) substructure evolution (the LABs and the HABs are displayed as red and black lines, LABs (2° ≤ θ < 15°) and HABs (θ ≥ 15°)), (**g**–**i**) frequency of misorientation distribution: (**a**,**d**,**g**) 30%, (**b**,**e**,**h**) 45%, (**c**,**f**,**i**) 75%.

Figure 18 shows the effects of deformation amount on the orientation distribution, substructure and misorientation distribution of the lamellar Ti65 alloy deformed at 980 °C and 0.01 s^−1^. As shown in Figure 18a, when the deformation amount is 30%, most α colonies still maintain relatively uniform orientations, such as colonies I–III. However, an obvious orientation gradient appears in colony IV, which may provide favorable conditions for subsequent dynamic spheroidization of lamellar α. When the deformation amount increases to 45%, the orientation gradients within colonies I–III become more pronounced, as shown in Figure 18b. Meanwhile, some spheroidized fine α grains are distributed along the edges of adjacent colonies, as marked by the red dashed ellipses. At a deformation amount of 75%, most lamellar α has undergone obvious dynamic spheroidization, and the degree of grain refinement is significantly enhanced, as shown in Figure 18c. Figure 18d–f show the corresponding grain-boundary maps, and Figure 18g–i present the misorientation distributions. At 30% deformation, most lamellar α remains unspheroidized, and low-angle grain boundaries are mainly located inside individual colonies. As the deformation amount increases to 45%, the fraction of low-angle grain boundaries decreases from 50.7% to 46.1%, while that of high-angle grain boundaries increases from 49.3% to 53.9%. These high-angle boundaries are mainly distributed within α colonies and along the edges of neighboring colonies, indicating that increasing deformation promotes α-lamella subdivision, boundary evolution and dynamic spheroidization.

It should be noted that, with increasing deformation amount, the fractions of boundaries near 10°, 60° and 90° in the misorientation distribution gradually decrease, whereas those in the ranges of 30–45° and 75–90° increase. This variation is closely related to the progressive destruction of the Burgers orientation relationship during dynamic spheroidization. At a low deformation amount, most lamellar α remains unspheroidized, and the crystallographic relationship inherited from the β → α transformation is largely retained. Therefore, the misorientation distribution at 30% deformation shows relatively high fractions near 10°, 60° and 90°. With further deformation, β-phase penetration and α-lamella fragmentation promote dynamic spheroidization. As a result, the spheroidized α particles no longer maintain a specific misorientation relationship with neighboring lamellar α, leading to increased fractions of misorientations in the ranges of 30–45° and 75–90°. When the deformation amount reaches 75%, the volume fraction of spheroidized α increases markedly, and the misorientation distribution becomes relatively uniform between 15° and 90° without distinct peaks. This further confirms that, with increasing deformation, the progressive fragmentation of the α lamellae and the increasing separation of α segments by the β phase reduce the crystallographic orientation coherence within the original α colonies inherited from the prior β phase.

Quantitative grain-boundary analysis was further performed using misorientation-angle criteria of 2–15° for LABs and ≥15° for HABs. At a deformation amount of 30%, the LAB and HAB fractions are 50.7% and 49.3%, respectively. With increasing deformation, the LAB fraction decreases whereas the HAB fraction increases, indicating progressive conversion of deformation-induced subboundaries into high-angle boundaries. This evolution is consistent with the accumulation and rearrangement of dislocations within the lamellar α phase, followed by subdivision of the α lamellae and progressive interfacial separation during dynamic spheroidization. At the highest deformation amount of 75%, the HAB fraction increased to 65.8% and together with the more dispersed misorientation distribution provides quantitative EBSD evidence for the enhanced subdivision and spheroidization of lamellar α. It should be distinguished that α-lamella fragmentation and spheroidization represent successive morphological stages rather than identical processes. Fragmentation refers to the subdivision or breakup of the original continuous α lamellae into shorter segments, while these segments may still retain an elongated morphology (k ≥ 2). k = l/b, where *I* and *b* represent the major and minor dimensions of an α particle, respectively. Dynamic spheroidization involves further interface migration and morphological adjustment, leading to isolated near-equiaxed α particles with k < 2. Therefore, only α particles satisfying the aspect-ratio criterion were included in the quantitatively determined spheroidized fraction.

**Figure 19:**
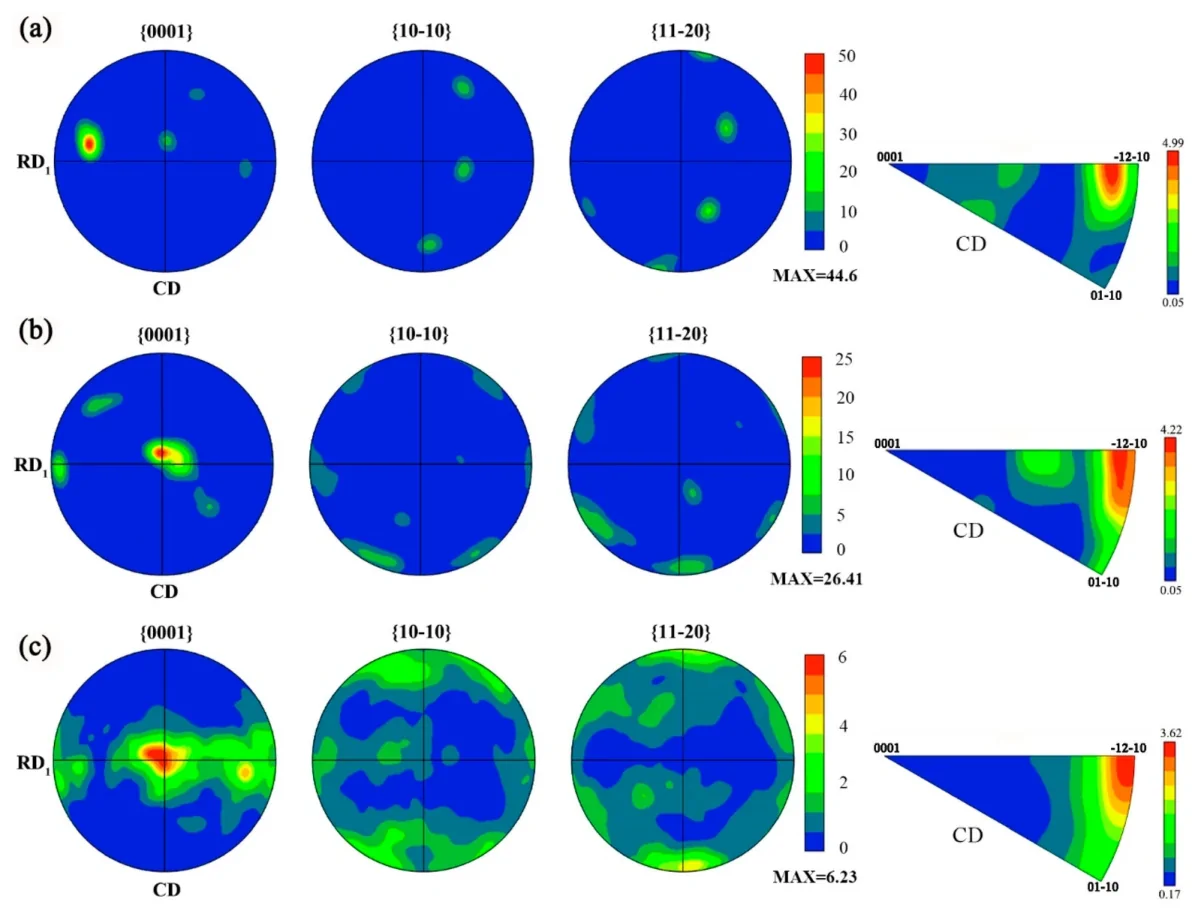
The texture evolution of the α phase of lamellar microstructure under parameters of 980 °C and 0.01 s^−1^ with different deformation amounts: (**a**) 30%, (**b**) 45%, (**c**) 75%.

Figure 19 shows the pole figures and inverse pole figures of the lamellar Ti65 alloy deformed at 980 °C and 0.01 s^−1^ under different deformation amounts. At 30% deformation, the {0001} pole figure in Figure 19a shows a relatively concentrated orientation distribution. This is mainly because most α lamellae still retain their colony-like morphology, and the α lamellae within the same colony have similar crystallographic orientations. Only local orientation gradients are formed at this deformation amount. Therefore, a relatively high maximum pole density is obtained, and the inverse pole figure indicates that the dominant orientation is close to <11-20>//CD. When the deformation amount increases to 45%, the orientation gradient within α colonies becomes more pronounced. As a result, the orientations within the same colony become more dispersed, leading to a clear decrease in the maximum pole density, as shown in Figure 19b. Meanwhile, the fiber texture gradually spreads from <11-20>//CD toward <10-10>//CD and <-24-23>//CD. At 75% deformation, the c-axis of the deformed α phase tends to rotate away from the compression direction, and the {0001} pole figure exhibits a more dispersed basal-pole distribution, as shown in Figure 19c. The maximum pole density decreases to 6.23 mrd. This indicates that extensive dynamic spheroidization not only promotes grain refinement but also weakens the initial texture intensity of the lamellar microstructure.

## 4. Conclusions

In this work, the hot deformation behavior, constitutive modeling, hot processing map and microstructure evolution of a novel near-α Ti65 titanium alloy with an initial lamellar microstructure were systematically investigated. The main conclusions are as follows:

(1) The flow stress of the lamellar Ti65 alloy decreases with increasing deformation temperature and decreasing strain rate. The flow-softening behavior is more pronounced in the α + β phase region than in the β single-phase region. The apparent activation energies for hot deformation are 1050.27 kJ/mol in the α + β region and 203.51 kJ/mol in the β region, indicating that the α + β deformation is controlled by complex microstructural evolution involving α-lamella kinking, fragmentation, spheroidization and phase-boundary migration, whereas dynamic recovery is the dominant mechanism in the β region, accompanied by limited β dynamic recrystallization.
(2) Strain-compensated Arrhenius-type constitutive models were established for the lamellar Ti65 alloy in different phase regions. The material constants show obvious strain dependence and can be well described by sixth-order polynomial functions. The established models exhibit good predictive accuracy, with R and AARE values of 0.98 and 5.97% in the α + β region and 0.99 and 4.29% in the β region, respectively, suggesting that the model prediction is slightly more accurate in the β single-phase region.
(3) Processing maps based on the dynamic materials model identify two instability domains at high strain rates: 990–1020 °C/3.5–10 s^−1^ in the upper α + β region and 1060–1110 °C/1.65–10 s^−1^ in the β region. Microstructural observations confirm that these instability domains are associated with heterogeneous α spheroidization, localized deformation, β dynamic recrystallization and abnormal β grain growth. Therefore, these domains should be avoided during hot processing of the Ti65 alloy with lamellar microstructure.
(4) Increasing deformation amount at 980 °C and 0.01 s^−1^ promotes α-lamella fragmentation, dynamic spheroidization and microstructural refinement. The lamellar microstructure gradually evolves from a bimodal-like morphology toward an equiaxed structure, while the transformed β fraction decreases and the spheroidized α fraction increases. EBSD results further show that increasing deformation also reduces texture intensity, with the maximum pole density decreasing to 6.23 mrd at a high deformation amount.

## Figures and Tables

**Table 1:** Chemical composition of as-forged Ti65 titanium alloy (wt. %).

Al	Sn	Zr	Ta	W	Si	Nb	Mo	C	Ti
5.92	3.94	3.45	0.86	0.66	0.48	0.32	0.31	0.05	Bal.

Note: the data tested by authors.

**Table 2:** The variables of lamellar microstructure determined using the polynomial fitting approach in the α + β phase region.

Material Parameters	Sixth-Order Polynomial Fitting
ln*A*	$\ln A\;=\;145.6\;-\;785.14\varepsilon \;+\;3302.38\varepsilon^{2}\;-\;7651.93\varepsilon^{3}\;+\;10,068.38\varepsilon^{4}\;-\;7128.97\varepsilon^{5}\;+\;2115.45\varepsilon^{6}$
*n*	$n\;=\;4.58\;-\;14.15\varepsilon \;+\;72.07\varepsilon^{2}\;-\;192.13\varepsilon^{3}\;+\;281.39\varepsilon^{4}\;-\;214.68\varepsilon^{5}\;+\;67.09\varepsilon^{6}$
*Q*	$Q\;=\;1542.43\;-\;8256.63\varepsilon \;+\;34,703.80\varepsilon^{2}\;-\;80.277.39\varepsilon^{3}\;+\;105,426.01\varepsilon^{4}\;-\;74,545.05\varepsilon^{5}\;+\;22,111.79\varepsilon^{6}$
*α*	$\alpha \;=\;0.01\;-\;0.01\varepsilon \;+\;0.15\varepsilon^{2}\;-\;0.44\varepsilon^{3}\;+\;0.70\varepsilon^{4}\;-\;0.60\varepsilon^{5}\;+\;0.21\varepsilon^{6}$

**Table 3:** The variables of lamellar microstructure determined using the polynomial fitting approach in the single β phase region.

Material Parameters	Sixth-Order Polynomial Fitting
ln*A*	$\ln A\;=\;33.09\;-\;328.92\varepsilon \;+\;2231.93\varepsilon^{2}\;-\;6867.05\varepsilon^{3}\;+\;10,762.49\varepsilon^{4}\;-\;8381.06\varepsilon^{5}\;+\;2574.67\varepsilon^{6}$
*n*	$n\;=\;3.78\;-\;9.07\varepsilon \;+\;44.78\varepsilon^{2}\;-\;116.92\varepsilon^{3}\;+\;166.44\varepsilon^{4}\;-\;122.56\varepsilon^{5}\;+\;36.65\varepsilon^{6}$
*Q*	$Q\;=\;397.85\;-\;3786.69\varepsilon \;+\;25,627.36\varepsilon^{2}\;-\;78,805.62\varepsilon^{3}\;+\;123,466.99\varepsilon^{4}\;-\;96,125.81\varepsilon^{5}\;+\;29,530.56\varepsilon^{6}$
*α*	$\alpha \;=\;0.03\;-\;0.11\varepsilon \;+\;0.72\varepsilon^{2}\;-\;2.08\varepsilon^{3}\;+\;3.23\varepsilon^{4}\;-\;2.59\varepsilon^{5}\;+\;0.84\varepsilon^{6}$

## Data Availability

The original contributions presented in this study are included in the article/Supplementary Materials. Further inquiries can be directed to the corresponding author.

## Supplementary Materials

The following supporting information can be downloaded at: https://www.mdpi.com/article/10.3390/ma19173749/s1, Table S1: Polynomial-order sensitivity analysis; Table S2: Leave-one-condition-out validation of different polynomial orders; Figure S1: Metallographic microstructure of Ti65 alloy after water quenching at different temperatures: (a) 1025 °C, (b) 1030 °C, (c) 1035 °C, (d) 1040 °C, (e) 1045 °C, (f) 1050 °C.

## References

[1] Banerjee D., Williams J.C. (2013). Perspectives on titanium science and technology. Acta Mater..

[2] Chatterjee P.P. (2016). Recent developments in aerospace materials. J. Metall. Mater. Sci..

[3] Xu S., Zhang H.M., Xiao N.M., Qiu R.S., Cui Z.S., Fu M.W. (2023). Mechanisms of macrozone elimination and grain refinement of near α Ti alloy via the spheroidization of the Widmannstätten structure. Acta Mater..

[4] Sun T., Deng Y., Liu W.H., Teng H.H., Wang R.Q., Sun C.Y., Deng H., Zhou J. (2024). Substructure and texture evolution of a novel near-α titanium alloy with bimodal microstructure during hot compression in α + β phase region. J. Alloys Compd..

[5] Sun T., Sun L., Teng H., Liu W., Wang R., Zhao X., Zhou J. (2024). Constitutive model and microstructure evolution of Ti65 titanium alloy. Materials.

[6] Gao P.F., Fu M.W., Zhan M., Lei Z.N., Li Y.X. (2020). Deformation behavior and microstructure evolution of titanium alloys with lamellar microstructure in hot working process: A review. J. Mater. Sci. Technol..

[7] Zhao Z.B., Zhang B.H., Sun H., Wang Q.J., Liu J.R., Yang R. (2023). Influence of globularization process on local texture evolution of a near-α titanium alloy with a transformed microstructure. Metall. Mater. Trans. A.

[8] Semiatin S.L. (2020). An overview of the thermomechanical processing of α/β titanium alloys: Current status and future research opportunities. Metall. Mater. Trans. A.

[9] Miller R.M., Bieler T.R., Semiatin S.L. (1999). Flow softening during hot working of Ti-6Al-4V with a lamellar colony microstructure. Scr. Mater..

[10] Semiatin S.L., Bieler T.R. (2001). The effect of alpha platelet thickness on plastic flow during hot working of TI–6Al–4V with a transformed microstructure. Acta Mater..

[11] Yadav P., Saxena K.K., Sehgal S., Singh T., Bahl S. (2023). Hot deformation behaviour of Ti alloys: A review on physical simulation and deformation mechanisms. Proc. Inst. Mech. Eng. Part E.

[12] Lehnert W., Cuong N.D. (1996). Experimental and mathematical simulation of microstructural evolution during hot rolling of Al and Cu material. J. Mater. Process. Technol..

[13] Yu H., Cao S., Youssef S.S., Ma Y.J., Lei J.F., Qi Y., Hu Q.M., Yang R. (2021). Generalized stacking fault energies and critical resolved shear stresses of random α-Ti-Al alloys from first-principles calculations. J. Alloys Compd..

[14] Tsuru T., Itakura M., Yamaguchi M., Watanabe C., Miura H. (2022). Dislocation core structure and motion in pure titanium and titanium alloys: A first-principles study. Comput. Mater. Sci..

[15] Ge J.Y., Zhan X.D., Li C., Zhang X.Y., Zhou K. (2023). Dynamic spheroidization mechanism and its orientation dependence of Ti-6Al-2Mo-2V-1Fe alloy during subtransus hot deformation. Materials.

[16] Zheng X.Y., Wang K., Zhang C., Xin R.L., Liu Q. (2022). Evolution mechanism of lamellar α and interlayered β during hot compression of TC21 titanium alloy with a widmanstätten structure. Chin. J. Aeronaut..

[17] Zhang J., Li H.W., Zhan M. (2020). Review on globularization of titanium alloy with lamellar colony. Manuf. Rev..

[18] Buzolin R.H., Lasnik M., Krumphals A., Poletti M.C. (2021). Hot deformation and dynamic α-globularization of a Ti-17 alloy: Consistent physical model. Mater. Des..

[19] Wang Q.R., Wang Q.J., Wang K.S., Wang W., Zhang P.H., Wang X., Zhang P.P. (2026). The phase-dependent behavior of high-temperature deformation in Ti65 titanium Alloy: Flow softening, recrystallization mechanisms, and slip system evolution. J. Mater. Res. Technol..

[20] Zhu P., Yang S., Gao Z.J., Liu J., Zhou L. (2024). Optimization of hot deformation parameters for multi-directional forging of Ti65 alloy based on the integration of processing maps and finite element method. J. Mater. Res. Technol..

[21] Li C.M., Huang L., Zhao M.J., Guo S.Q., Su Y., Li J.J. (2022). Characterization of hot workability of Ti-6Cr-5Mo-5V-4Al alloy based on hot processing map and microstructure evolution. J. Alloys Compd..

[22] Zhang Z.X., Fan J.K., Tang B., Kou H.C., Wang J., Chen Z.Y., Li J.S. (2020). Microstructure/texture evolution maps to optimize hot deformation process of near-α titanium alloy. Prog. Nat. Sci. Mater. Int..

[23] Morakabati M., Hajari A. (2020). Hot working behavior of near alpha titanium alloy analyzed by mechanical testing and processing map. Trans. Nonferrous Met. Soc. China.

[24] Huang L., Li C.M., Li C.L., Hui S.X., Yu Y., Zhao M.J., Guo S.Q., Li J.J. (2022). Research progress on microstructure evolution and hot processing maps of high strength β titanium alloys during hot deformation. Trans. Nonferrous Met. Soc. China.

[25] Su Y., Kong F.T., You F.H., Wang X.P., Chen Y.Y. (2020). The high-temperature deformation behavior of a novel near-α titanium alloy and hot-forging based on the processing map. Vacuum.

[26] Zhang Z.X., Fan J.K., Tang B., Kou H.C., Wang J., Wang X., Wang S.Y., Wang Q.J., Yong C.Z., Li J.S. (2020). Microstructural evolution and FCC twinning behavior during hot deformation of high temperature titanium alloy Ti65. J. Mater. Sci. Technol..

[27] Yue K., Liu J.R., Zhang H.J., Yu H., Song Y., Hu Q., Wang Q., Yang R. (2020). Precipitates and alloying elements distribution in near α titanium alloy Ti65. J. Mater. Sci. Technol..

[28] Hu L., Lang M.W., Shi L.X., Li M., Zhou T., Bao C.L., Yang M.B. (2023). Study on hot deformation behavior of homogenized Mg-8.5Gd-4.5Y-0.8Zn-0.4Zr alloy using a combination of strain-compensated Arrhenius constitutive model and finite element simulation method. J. Magnes. Alloys.

[29] Basanth K.K., Saxena K.K., Dey S.R., Pancholi V., Bhattacharjee A. (2017). Processing map-microstructure evolution correlation of hot compressed near alpha titanium alloy (TiHy 600). J. Alloys Compd..

[30] Gao P.F., Zhan M., Fan X.G., Lei Z.N., Cai Y. (2017). Hot deformation behavior and microstructure evolution of TA15 titanium alloy with nonuniform microstructure. Mater. Sci. Eng. A.

[31] Xu X.Y., Zhang B.H., Xue J.Y., Li F., Su S., Chang H. (2024). Microstructural characteristics and recrystallization mechanism of Ti-6.5Al-2Zr-1Mo-1V alloy during two-stage hot deformation. J. Mater. Res. Technol..

[32] Saxena K.K., Pancholi V., Jha S.K., Chaudhari G.P., Srivastava D., Dey G.K. (2017). A novel approach to understand the deformation behavior in two phase region using processing map. J. Alloys Compd..

[33] Syed F.W., Jaladurgam N.R., Kumar V.A., Gupta R.K., Kanjarla A.K. (2021). Hot deformation characteristics and microstructure evolution of Ti–5Al–3Mo–1.5V alloy. Int. J. Adv. Eng. Sci. Appl. Math..

[34] Hao F., Xiao J.F., Feng Y., Wang Y., Ju J.T., Du Y.X., Wang K.X., Xue L.N., Nie Z.H., Tan C.W. (2020). Tensile deformation behavior of a near-α titanium alloy Ti-6Al-2Zr-1Mo-1V under a wide temperature range. J. Mater. Res. Technol..

[35] Jia W.J., Zeng W.D., Zhou Y.G., Liu J.R., Wang Q.J. (2011). High-temperature deformation behavior of Ti60 titanium alloy. Mater. Sci. Eng. A.

[36] Chen W., Zeng W.D., Xu J.W., Zhou D.D., Wang S.M., He S.T. (2019). Deformation behavior and microstructure evolution during hot working of Ti60 alloy with lamellar starting microstructure. J. Alloys Compd..

[37] Yu Y., Xiong B.Q., Hui S.X., Ye W.J. (2013). Hot deformation behavior and globularization mechanism of Ti-6Al-4V-0.1B alloy with lamellar microstructure. Rare Met..

[38] Wen D.X., Yue T.Y., Xiong Y.B., Wang K., Wang J.K., Zheng Z.Z., Li J.J. (2021). High-temperature tensile characteristics and constitutive models of ultrahigh strength steel. Mater. Sci. Eng. A.

[39] Wang Y.T., Li J.B., Xin Y.C., Li C.Z., Cheng Y., Chen X.H., Rashad M., Liu B., Liu Y. (2019). Effect of Zener-Hollomon parameter on hot deformation behavior of CoCrFeMnNiC0.5 high entropy alloy. Mater. Sci. Eng. A.

[40] Raj R. (1981). Development of a processing map for use in warm-forming and hot-forming processes. Metall. Trans. A.

[41] Prasad Y.V., Gegel H.L., Doraivelu S.M., Malas J.C., Morgan J.T., Lark K.A., Barker D.R. (1984). Modeling of dynamic material behavior in hot deformation: Forging of Ti-6242. Metall. Trans. A.

[42] Son K.T., Kim M.H., Kim S.W., Lee J.W., Hyun S.K. (2018). Evaluation of hot deformation characteristics in modified AA5052 using processing map and activation energy map under deformation heating. J. Alloys Compd..

[43] Lukaszek-Solek A., Krawczyk J., Sleboda T., Grelowski J. (2019). Optimization of the hot forging parameters for 4340 steel by processing maps. J. Mater. Res. Technol..

[44] Prasad Y.V., Seshacharyulu T. (1998). Modelling of hot deformation for microstructural control. Int. Mater. Rev..

[45] Prasad Y.V. (2003). Processing maps: A status report. J. Mater. Eng. Perform..

[46] Mirahmadi D., Dehghani K., Shamsipur A., Kalaki A. (2023). Hot deformation behavior, microstructure evolution and processing map of Cu–2Be alloy. J. Mater. Res. Technol..

[47] Zhou X., Wang K.L., Lu S.Q., Li X., Feng R., Zhong M.J. (2020). Flow behavior and 3D processing map for hot deformation of Ti-2.7Cu alloy. J. Mater. Res. Technol..

