# Supplementary material for: Hot Deformation Behavior and Microstructure Evolution of a Novel Near-α Titanium Alloy with Initial Lamellar Microstructure During Hot Compression
Source: Materials (Basel). 2026 Sep 3;19(17):3749. doi: 10.3390/ma19173749 (PMC13567099; doi:10.3390/ma19173749)
Supplement: Supplementary file 1 [file materials-19-03749-s001.zip › materials-4506388-supplementary.pdf]

Supplementary Information

Table S1: Polynomial-order sensitivity analysis

Phase region	Parameter	Polynomial order	R^2	Adjusted R^2	RMSE
α+β	$\ln A$	2	0.915360	0.903268	2.847098
		3	0.970127	0.963234	1.691419
		4	0.997065	0.996086	0.530206
		5	0.998546	0.997884	0.373217
		**6**	**0.998976**	**0.998362**	**0.313113**
	n	2	0.854500	0.833714	0.032113
		3	0.870599	0.840738	0.030284
		4	0.987657	0.983542	0.009353
		5	0.993527	0.990585	0.006773
		6	0.999382	0.999011	0.002093
	Q	2	0.915291	0.903190	29.925985
		3	0.969620	0.962609	17.921640
		4	0.997130	0.996173	5.508735
		5	0.998606	0.997972	3.839153
		**6**	**0.999032**	**0.998451**	**3.198772**
	α	2	0.997625	0.997286	0.000111
		3	0.997840	0.997342	0.000106
		4	0.999679	0.999571	0.000041
		5	0.999730	0.999607	0.000038
		**6**	**0.999809**	**0.999694**	**0.000032**
β	$\ln A$	2	0.844872	0.822711	0.481955
		3	0.859957	0.827639	0.457923
		4	0.881577	0.842102	0.421095
		5	0.948343	0.924863	0.278115
		**6**	**0.989152**	**0.982643**	**0.127448**
	n	2	0.900400	0.886171	0.022139
		3	0.942847	0.929658	0.016771
		4	0.986174	0.981566	0.008249
		5	0.993644	0.990755	0.005593

	**6**	**0.996160**	**0.993857**	**0.004347**
	2	0.817916	0.791904	5.594593
	3	0.838177	0.800833	5.274144
Q	4	0.864690	0.819587	4.822772
	5	0.941157	0.914410	3.180396
	**6**	**0.987921**	**0.980673**	**1.440955**
	2	0.986862	0.984985	0.000299
	3	0.990323	0.988090	0.000257
α	4	0.998593	0.998124	0.000098
	5	0.998762	0.998199	0.000092
	**6**	**0.999716**	**0.999546**	**0.000044**

Table S2: Leave-one-condition-out validation of different polynomial orders

Phase region	Polynomial order	R_{CV}	$AARE_{CV}$ (%)	$RMSE_{CV}$ (MPa)
	2	0.988961	8.7088	7.1023
	3	0.989130	8.7241	7.0573
α+β	4	0.989245	8.7014	7.0280
	5	0.989248	8.6916	7.0270
	6	0.989250	8.6914	7.0262
	2	0.994347	5.7236	2.6930
	3	0.994803	5.7028	2.5861
β	4	0.995025	5.6688	2.5307
	5	0.995032	5.6834	2.5289
	6	0.995049	5.6940	2.5244

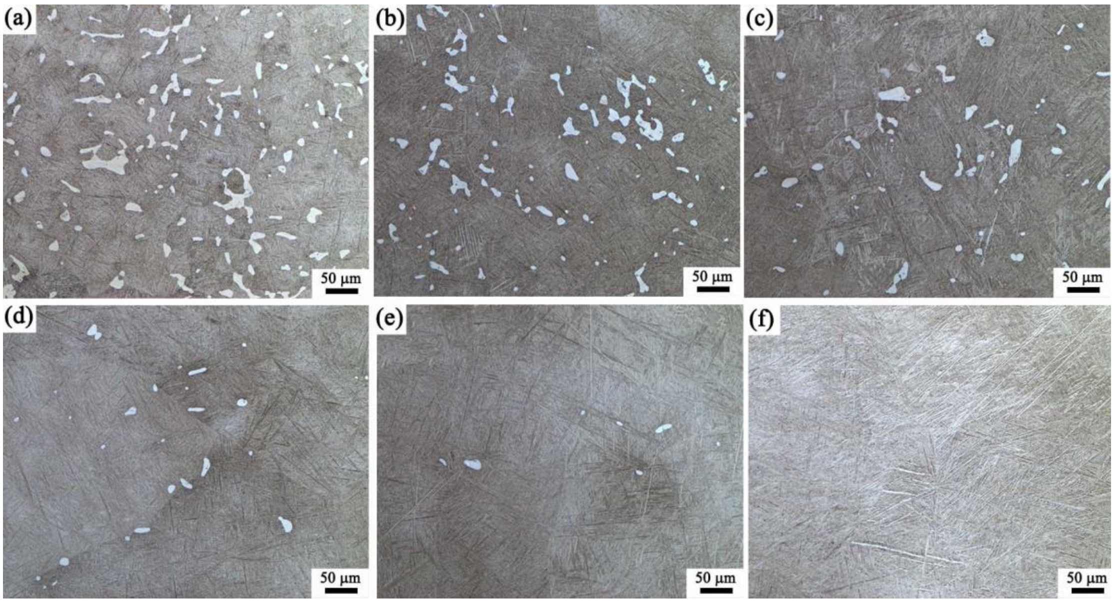

Figure S1: Metallographic microstructure of Ti65 alloy after water quenching at different temperatures: (a) 1025 ℃, (b) 1030 ℃, (c) 1035 ℃, (d) 1040 ℃, (e) 1045 ℃, (f) 1050 ℃
